# Fine‐Mapping the Results From Genome‐Wide Association Studies of Primary Biliary Cholangitis Using SuSiE and h2‐D2

**DOI:** 10.1002/gepi.22592

**Published:** 2024-10-06

**Authors:** Aida Gjoka, Heather J. Cordell

**Affiliations:** ^1^ Population Health Sciences Institute Newcastle University Newcastle upon Tyne UK

**Keywords:** genetic fine‐mapping, PBC, summary statistics, variable selection

## Abstract

The main goal of fine‐mapping is the identification of relevant genetic variants that have a causal effect on some trait of interest, such as the presence of a disease. From a statistical point of view, fine mapping can be seen as a variable selection problem. Fine‐mapping methods are often challenging to apply because of the presence of linkage disequilibrium (LD), that is, regions of the genome where the variants interrogated have high correlation. Several methods have been proposed to address this issue. Here we explore the ‘Sum of Single Effects’ (SuSiE) method, applied to real data (summary statistics) from a genome‐wide meta‐analysis of the autoimmune liver disease primary biliary cholangitis (PBC). Fine‐mapping in this data set was previously performed using the FINEMAP program; we compare these previous results with those obtained from SuSiE, which provides an arguably more convenient and principled way of generating ‘credible sets’, that is set of predictors that are correlated with the response variable. This allows us to appropriately acknowledge the uncertainty when selecting the causal effects for the trait. We focus on the results from SuSiE‐RSS, which fits the SuSiE model to summary statistics, such as z‐scores, along with a correlation matrix. We also compare the SuSiE results to those obtained using a more recently developed method, h2‐D2, which uses the same inputs. Overall, we find the results from SuSiE‐RSS and, to a lesser extent, h2‐D2, to be quite concordant with those previously obtained using FINEMAP. The resulting genes and biological pathways implicated are therefore also similar to those previously obtained, providing valuable confirmation of these previously reported results. Detailed examination of the credible sets identified suggests that, although for the majority of the loci (33 out of 56) the results from SuSiE‐RSS seem most plausible, there are some loci (5 out of 56 loci) where the results from h2‐D2 seem more compelling. Computer simulations suggest that, overall, SuSiE‐RSS generally has slightly higher power, better precision, and better ability to identify the true number of causal variants in a region than h2‐D2, although there are some scenarios where the power of h2‐D2 is higher. Thus, in real data analysis, the use of complementary approaches such as both SuSiE and h2‐D2 is potentially warranted.

## 1 Introduction

Primary biliary cholangitis (PBC), formerly known as primary biliary cirrhosis, is an autoimmune liver disease that results from a combination of genetic and environmental risk factors. The largest genome‐wide association study (GWAS) performed to date in PBC was an international genome‐wide meta‐analysis involving five European and two East Asian cohorts (Cordell et al. 2021), which identified 57 genome‐wide significant loci (21 novel), including 47 loci that were significantly implicated within the European cohorts. Fine‐mapping using the FINEMAP package (Benner et al. 2016) was performed within each genome‐wide significant risk locus to identify independent associations and to construct ‘credible sets’ of variants most likely to be causal in PBC. At most loci the association signal was best explained by a single variant, but at 16 of the 47 European‐identified loci it was best explained by ≥2 independent variants. Cordell et al. (2021) performed the fine mapping separately within the European and East Asian cohorts. In principle, combining the European with the East Asian cohorts should provide greater power and resolution for fine‐mapping (on account of both the larger sample size and the differing LD patterns between Europeans and East Asians). In practice, however, this proved problematic on account of both the differing LD patterns and the different genotyping chips used for the different cohorts, resulting in a limited overlap of variants that passed postimputation QC. For the current work, therefore, we focus on fine‐mapping the results obtained from the European cohorts, which provides the larger sample size and a higher resolution (in terms of the number of variants interrogated) than can be obtained by combining the results from the European and East Asian cohorts.

Genome‐wide association studies (GWAS), such as those performed in PBC, have been highly effective for identifying genetic variants—usually single‐nucleotide polymorphisms (SNPs)—that are associated with complex traits (Schaid, Chen, and Larson 2018). A large number of variants have been associated with both rare and common genetic disorders. While numerous variants are reported as being causally associated with complex traits, false assignments of causality at the variant level are an important issue (MacArthur et al. 2014). Assigning causality can be challenging due to the presence of linkage disequilibrium (LD) across the genome, that is, SNPs within a particular genomic region that exhibit a high correlation structure. A frequently used approach to address this issue involves the use of fine‐mapping techniques (Spain and Barrett 2015). Fine‐mapping methods aim to identify genetic variants that causally affect some trait of interest. Fine mapping can be framed as a variable selection problem. Given the high number of variables and their correlation structure, an attractive approach is to use Bayesian methods. Uffelmann et al. (2021) provide an excellent review on several fine‐mapping techniques used to optimise the selection of variables by considering a Bayesian approach. A variety of different methods and software packages have been developed for this purpose, including CAVIAR (Hormozdiari et al. 2014), PAINTOR (Kichaev et al. 2014), CAVIARBF (Chen et al. 2015), FINEMAP (Benner et al. 2016), JAM (Newcombe, Conti, and Richardson 2016), DAP (Wen et al. 2016), SuSiE (Wang et al. 2020) and SuSiE‐RSS (Zou et al. 2022).

Here, we consider the recently proposed fine‐mapping method based on summary statistics, SuSiE‐RSS. SuSiE‐RSS is an extension of the original SuSiE (‘Sum of Single Effects’) model introduced by Wang et al. (2020), where ‘RSS’ signifies ‘regression with summary statistics’. SuSiE addresses the fine‐mapping problem by writing the vector of regression coefficients as a sum of ‘single‐effect’ vectors, each with one nonzero element, and then performing a Bayesian analogue of stepwise selection. The end result is the generation of one or more ‘credible sets’ of variables that encompass the uncertainty about which variables should be selected as causal, when multiple, highly correlated variables compete with each other. SuSiE and SuSiE‐RSS were shown to perform competitively with alternative competing methods such as FINEMAP (Benner et al. 2016) and CAVIAR (Hormozdiari et al. 2014), while providing an arguably more convenient and principled way of generating credible sets of putative causal variants.

While our work was in progress, a new method, h2‐D2 (Li, Sham, and Zhang 2024), which uses a continuous global‐local shrinkage prior (in contrast to the discrete mixture prior used by previous methods) was published. We therefore further compare our results from SuSIE‐RSS with those from h2‐D2, which uses the same inputs as SuSiE‐RSS, and was shown in some cases (Li, Sham, and Zhang 2024) to outperform SuSiE‐RSS.

## 2 Methods

### 2.1 Application to Real PBC Data

We applied SuSiE‐RSS to summary statistics derived from logistic regression analysis of the European data from the GWAS of Cordell et al. (2021) with a total sample size of 24,510 individuals (8021 cases and 16,489 controls). We focussed on variants with MAF>0.0001 lying within 56 of the 57 genome‐wide significant loci identified by Cordell et al. (2021), excluding the human leucocyte antigen (HLA) region on account of its extended LD and complicated correlation structure (A more detailed investigation of HLA based on a subset of the European data used here was previously performed by Darlay et al. [2018]). The 56 loci considered here were previously interrogated by Cordell et al. (2021) using FINEMAP. Cordell et al. (2021) previously used a complicated strategy involving ‘double genomic control’ (Devlin and Roeder 1999) in the contributing cohorts to derive their original summary statistics. Here, taking the view that these loci have already been established, we used a simpler strategy of re‐analysing the imputed genotype data from the combined European cohorts using logistic regression implemented in the software package PLINK (Purcell et al. 2007), with 10 genetic principal components included as covariates.

We built the correlation matrix for each locus based on individual‐level data from our own European samples, again using the software package PLINK. Where necessary, the correlation matrix R was enforced to be positive definite through use of the ‘repairMatrix(R, eps = 0.0001)’ function from the R package NMOF. We chose to use the in‐sample correlation matrix after initial explorations using a covariance matrix derived from an external reference panel (1000 Genomes European‐ancestry individuals) generated a number of instances where the SuSiE‐RSS algorithm failed to converge, presumably because of discrepancies between the LD structure of the reference panel and our own European individuals. We then used the summary statistics (regression coefficients together with their corresponding standard errors) and the obtained correlation matrix to fit SuSiE‐RSS using the susieR R package. The coverage level threshold (i.e., the proportion of credible sets that should contain an effect variable) was set to be 0.95, except for four loci where this threshold had to be lowered to achieve any nonempty credible sets: 2q21.3 where the level was specified to be 0.6, 9q32 and 11q24.3 where the level was specified to be 0.7 and 14q13.2 where the level was specified to be 0.8. Following visual inspection of our results, we also lowered the coverage threshold for locus 11p15.5 to 0.7, to force SuSiE‐RSS to identify the primary signal in this region that had been identified by Cordell et al. (2021).

While this work was in progress, a new Bayesian fine‐mapping method, h2‐D2 (Li, Sham, and Zhang 2024), which uses the same inputs as SuSiE‐RSS, and was shown in some cases to outperform SuSiE‐RSS, was published. We therefore repeated our analysis of the 56 loci using h2‐D2 (version 1.1, downloaded 12 March 2024), and compared the results obtained to those obtained from SuSiE. We initially used the default h2‐D2 coverage level threshold of 0.95, except for the five loci where the threshold had been lowered for SuSiE‐RSS, where we used the same lower threshold as had been used in SuSiE‐RSS. We subsequently had to reduce the h2‐D2 coverage threshold used to 0.9 for four loci (2p25.1, 4q24[2], 6q27 and 8q24.21), in order for h2‐D2 to generate any credible sets.

### 2.2 Initial Simulation Studies

For initial simulation studies, we used HAPGEN2 version 2.0.1 (Su, Marchini, and Donnelly 2011) to simulate data under three different scenarios, generating 100 replicates of 500 cases and 500 controls in each scenario. As a generating panel, we used the CEU HapMap genotypes in the region from 26.0 to 26.4 Mb of chromosome 21. In Scenario 1, only one risk variant was assumed to exist in the region, in Scenario 2 two risk variants were assumed, located relatively near to one another in a highly correlated subregion, and for Scenario 3, we chose two risk variants that were situated a slightly larger distance away from each other. The genotypic relative risks were assumed to be 2 and 4 for one and two copies of the risk allele respectively; we took the view that using a relatively small sample size with relatively large genotypic relative risks would produce summary statistics similar to those seen in larger cohorts at loci with smaller effect sizes, while being considerably quicker to simulate. Each simulation replicate was analysed in the same way as had been used for the PBC data. To evaluate the performance of SuSiE‐RSS and h2‐D2 we considered different metrics, such as the power (the number of times that each causal variant had been correctly detected within the generated credible set[s]), and the average number and size of credible sets, along with their variability as measured through their standard deviations.

### 2.3 Tailored Simulation Study Based on PBC Data

We conducted more extensive/realistic simulations using the genotyping data and association results from the PBC data set. The top associated variant from each credible set identified by either SuSiE‐RSS or h2‐D2 (as reported in Table S1) was assumed to be a true causal variant, with effect size (log odds ratio) determined from the real data analysis. A simulated binary (disease) phenotype was generated for each of the 24,510 individuals from the PBC study, with the probability of an individual being diseased determined based on their known genotypes at the causal variants (along with the assumed log odds ratios). The resulting ‘polygenic risk score’ for each individual was converted to a probability of being diseased using the logit function, which was then used as a threshold for determining case/control status (when applied to the output of the runif() function in R), with the threshold scaled to produce on average the same number of cases and controls as seen in the real PBC data set. The generated cases and controls were analysed via logistic regression in PLINK, and SuSiE‐RSS and h2‐D2 were applied to the resulting summary statistics, in the same way as in the real case/control analysis. The process was repeated for 100 simulation replicates (each generating different case/control phenotype designations) and the overall performance of SuSiE‐RSS and h2‐D2 in terms of identification of the true causal variants, and identification of the true number of causal variants in each credible set, was evaluated.

## 3 Results

### 3.1 Results From Application to PBC Data

Table 1 shows a comparison of the results obtained from SuSiE‐RSS and h2‐D2 with those previously obtained using FINEMAP. The FINEMAP results are shown as the posterior probabilities for the number of causal variants (columns 2‐4), while SuSiE‐RSS and h2‐D2 results are shown in terms of number of credible sets for each risk locus and the corresponding number of elements in each credible set (columns 5‐8). Overall we find generally good concordance between SuSiE‐RSS, h2‐D2 and FINEMAP in terms of the number of credible sets (i.e., number of putative causal variants) identified within each locus, except for a few cases where h2‐D2 gives an unusually large number of credible sets. However, similar to what was seen by Li, Sham, and Zhang (2024), the sizes of the credible sets generated by h2‐D2 are generally larger than those from SuSIE‐RSS.

**Table 1:** Results obtained from SuSiE‐RSS and h2‐D2 in comparison with previously obtained posterior probabilities from FINEMAP.

		FINEMAP posterior probabilities			SuSiE results		h2‐D2 results	
Locus number	Locus	1 variant	2 variants	3 variants	No of CS	CS sizes	No of CS	CS sizes
1	1p36.32	0.95	0.05	0	1	65	1	79
2	1p31.3	0.05	0.45	0.5	2	1, 4	1	1
3	1p13.1	0.95	0.05	0	1	29	1	29
4	1q23.1	0.92	0.08	0	1	33	1	48
5	1q31.3	0.8	0.19	0.01	1	6	1	20
6	1q32.1	0.85	0.14	0	1	33	1	37
7	2p25.1	0.88	0.12	0	1	7	1	11
8	2p23.3	0.1	0.8	0.09	2	2, 8	1	8
9	2q21.3	0.88	0.12	0	1	12	5	3, 2, 9, 32, 35
10	2q32.2	0	0	1	4	1, 14, 32, 19	3	1, 18, 33
11	2q33.2	0.61	0.37	0.02	1	39	1	54
12	3p24.3	0.88	0.11	0	1	14	1	25
13	3p24.2	0.91	0.02	0	1	10	1	13
14	3q13.33	0.8	0.17	0.02	1	2	1	3
15	3q25.33	0	0	1	4	1, 4, 11, 39	4	1, 4, 10, 45
16	4q24(1)	0.71	0.27	0.02	1	63	1	76
17	4q24(2)	0.9	0.1	0	1	23	1	48
18	5p13.2	0.85	0.15	0	1	15	1	16
19	5q21.1	0.96	0.04	0	1	87	3	1, 1, 126
20	5q31.3	0.92	0.08	0	1	61	1	66
21	5q33.3	0.81	0.18	0.01	1	1	1	10
22	6q21	0.24	0.64	0.12	2	11, 5	1	15
23	6q23.3	0.06	0.4	0.54	1	7	1	7
24	6q27	0.89	0.11	0	1	10	1	44
25	7p21.1	0.79	0.2	0.01	1	27	1	48
26	7p14.2‐p14.1	0.91	0.09	0	1	33	1	40
27	7q32.1	0	0.89	0.11	2	20, 6	2	10, 21
28	7q34	0.82	0.17	0.01	1	60	1	63
29	8q24.21	0.14	0.57	0.29	1	9	1	16
30	9q22.33	0.94	0.06	0	1	15	1	16
31	9q32	0.55	0.44	0.02	1	6	2	8, 38
32	10q11.23	0.66	0.07	0.27	1	1	1	19
33	11p15.5	0.01	0.9	0.09	2	1, 15	3	1, 14, 24
34	11q13.1	0.91	0.09	0	1	19	1	19
35	11q23.1	0.22	0.73	0.04	2	10, 57	2	38, 61
36	11q23.3	0.67	0.28	0.05	1	12	1	9
37	11q24.3	0.85	0.15	0	1	9	2	9, 97
38	12p13.31	0.89	0.1	0.01	1	1	1	1
39	12q24.12	0.88	0.12	0	1	3	2	3, 312
40	13q14.11	0.91	0.09	0	1	20	1	38
41	13q14.2‐q14.3	0.87	0.12	0.01	1	6	1	5
42	14q13.2	0.68	0.09	0.23	1	13	1	136
43	14q24.1	0.66	0.31	0.03	2	2, 62	1	3
44	14q32.12	0.47	0.48	0.05	2	18, 11	2	13, 17
45	14q32.32	0.39	0.54	0.08	1	3	5	1, 4, 3, 100, 2
46	16p13.13	0	0.6	0.41	2	15, 6	2	21, 10
47	16p12.1	0.92	0.07	0	1	10	1	10
48	16q22.1	0.94	0.06	0	1	65	1	94
49	16q24.1	0.24	0.63	0.13	1	2	1	2
50	17q12	0.96	0.04	0	1	24	1	23
51	17q21.31	0.99	0.01	0	1	1909	1	746
52	18q22.2	0.91	0.09	0	1	24	1	24
53	19p13.2	0.78	0.2	0.02	1	2	1	2
54	19p13.11	0.82	0.17	0.01	1	23	1	34
55	19q13.33	0	0.93	0.07	2	4, 6	2	5, 4
56	22q13.1	0	0.59	0.41	3	4, 22, 11	2	4, 30

*Note:* No of CS is the number of credible sets generated. CS sizes are the sizes of the generated credible sets.

Plots showing the results from application of SuSiE‐RSS and h2‐D2 to four example risk loci are shown in Figures 1, 2, 3, 4. Results for the full set of 56 risk loci are shown in Supporting Information S1: Figures S1–S19. The scatter‐plots show the obtained transformed *p*‐values for the SNPs in each locus, where the horizontal axis shows the position in Mb and the vertical axis shows the corresponding negative log‐transformed *p*‐value. The obtained credible sets are shown in coloured dots (red for the first credible set, blue for the second, and so on), with SNPs that were not selected to be part of any credible sets shown in grey.

**Figure 1:**
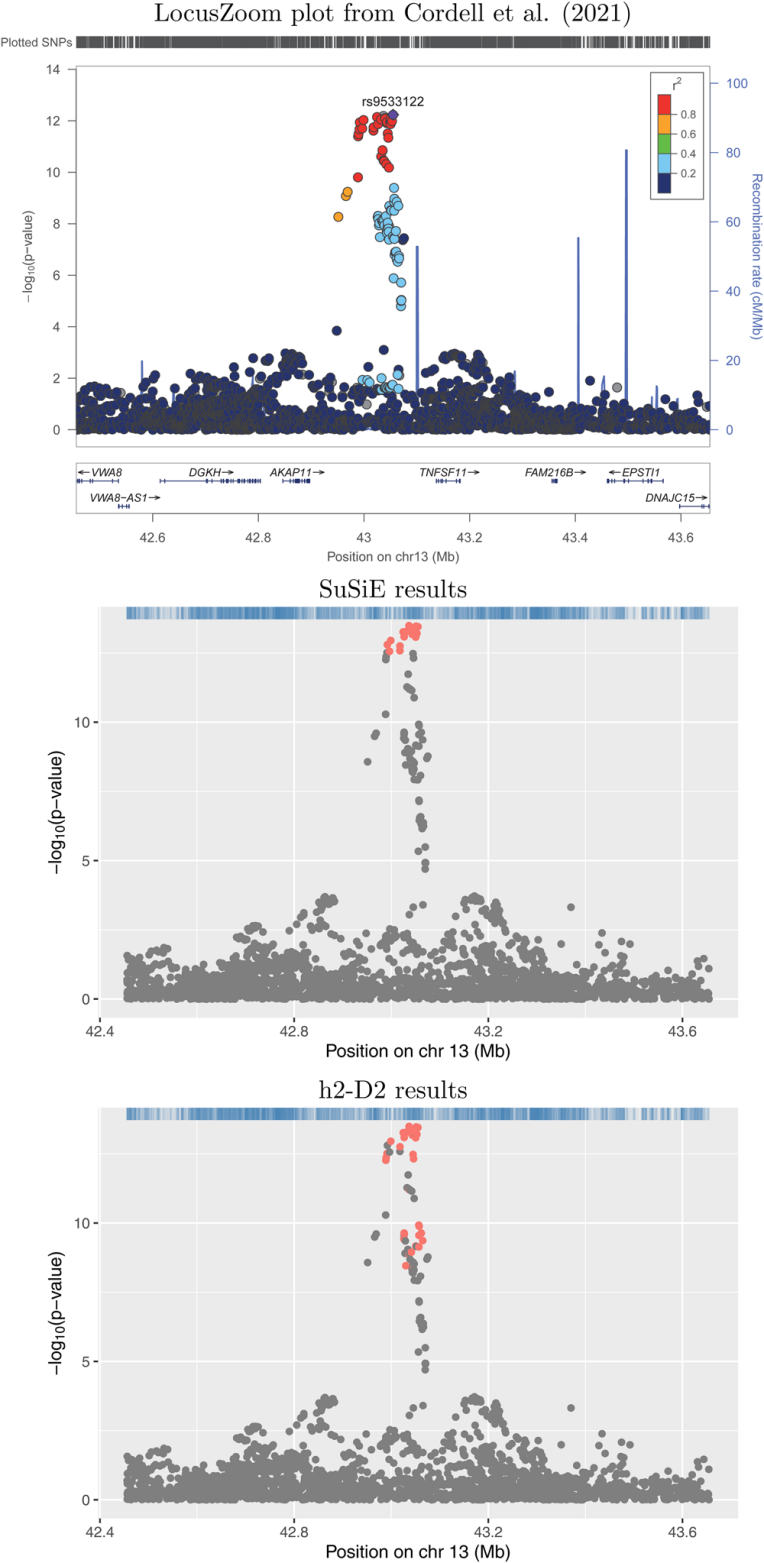
Association plots for 13q14.11. The *x*‐axis shows the base pair position and the *y*‐axis shows the −log10 *p*‐value of each SNP tested for association by Cordell et al. (2021) (top plot) or by using logistic regression (middle and lower plots); the summary statistics from the logistic regression analyses were used as input to SuSiE‐RSS and h2‐D2. SNPs in different credible sets from SuSiE (middle plot) and h2‐D2 (lower plot) are indicated in different colours. SNPs coloured in grey were not chosen as part of any credible set.

**Figure 2:**
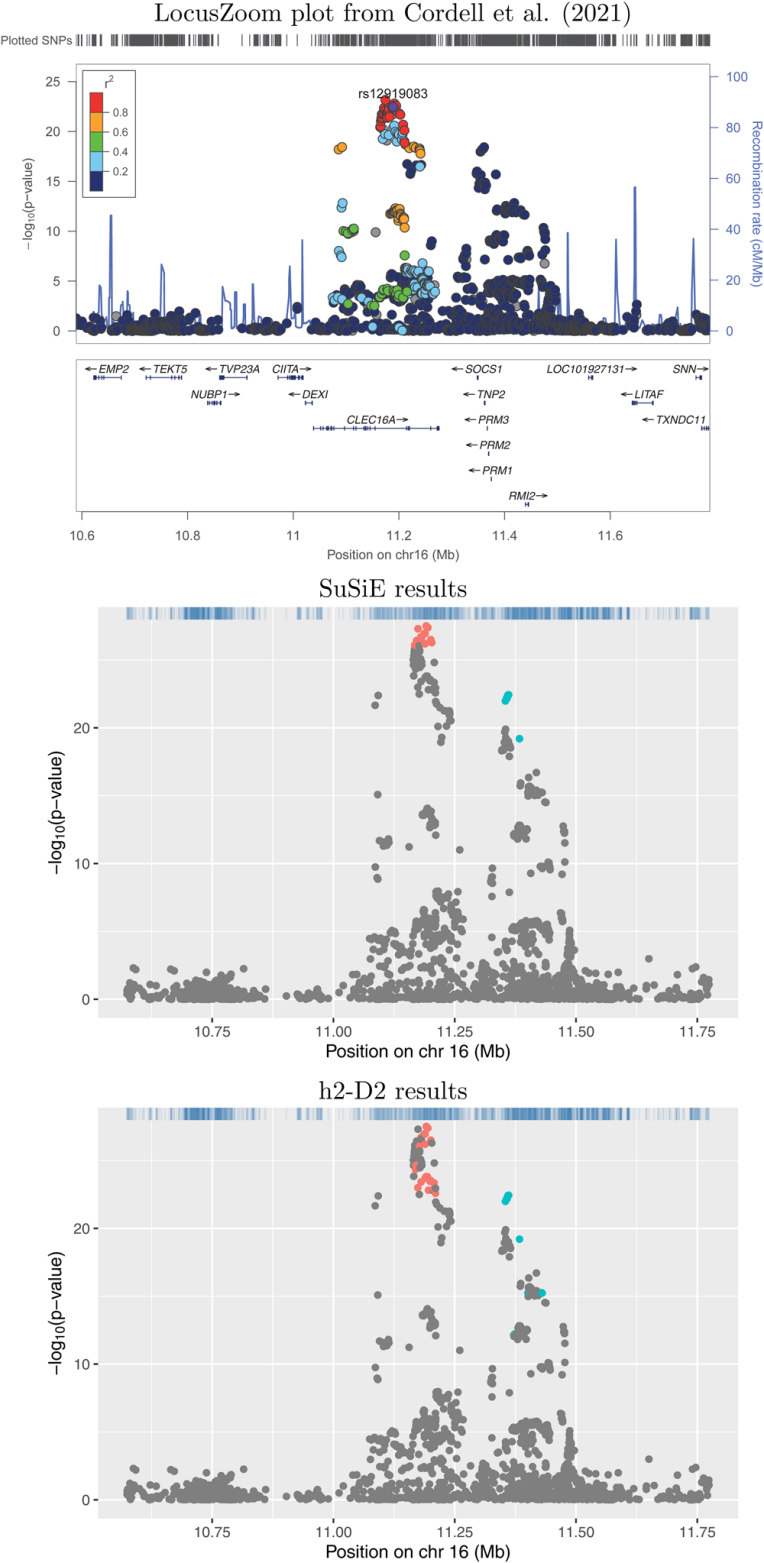
Association plots for 16p13.13. The *x*‐axis shows the base pair position and the *y*‐axis shows the −log10 *p*‐value of each SNP tested for association by Cordell et al. (2021) (top plot) or by using logistic regression (middle and lower plots); the summary statistics from the logistic regression analyses were used as input to SuSiE‐RSS and h2‐D2. SNPs in different credible sets from SuSiE (middle plot) and h2‐D2 (lower plot) are indicated in different colours. SNPs coloured in grey were not chosen as part of any credible set.

**Figure 3:**
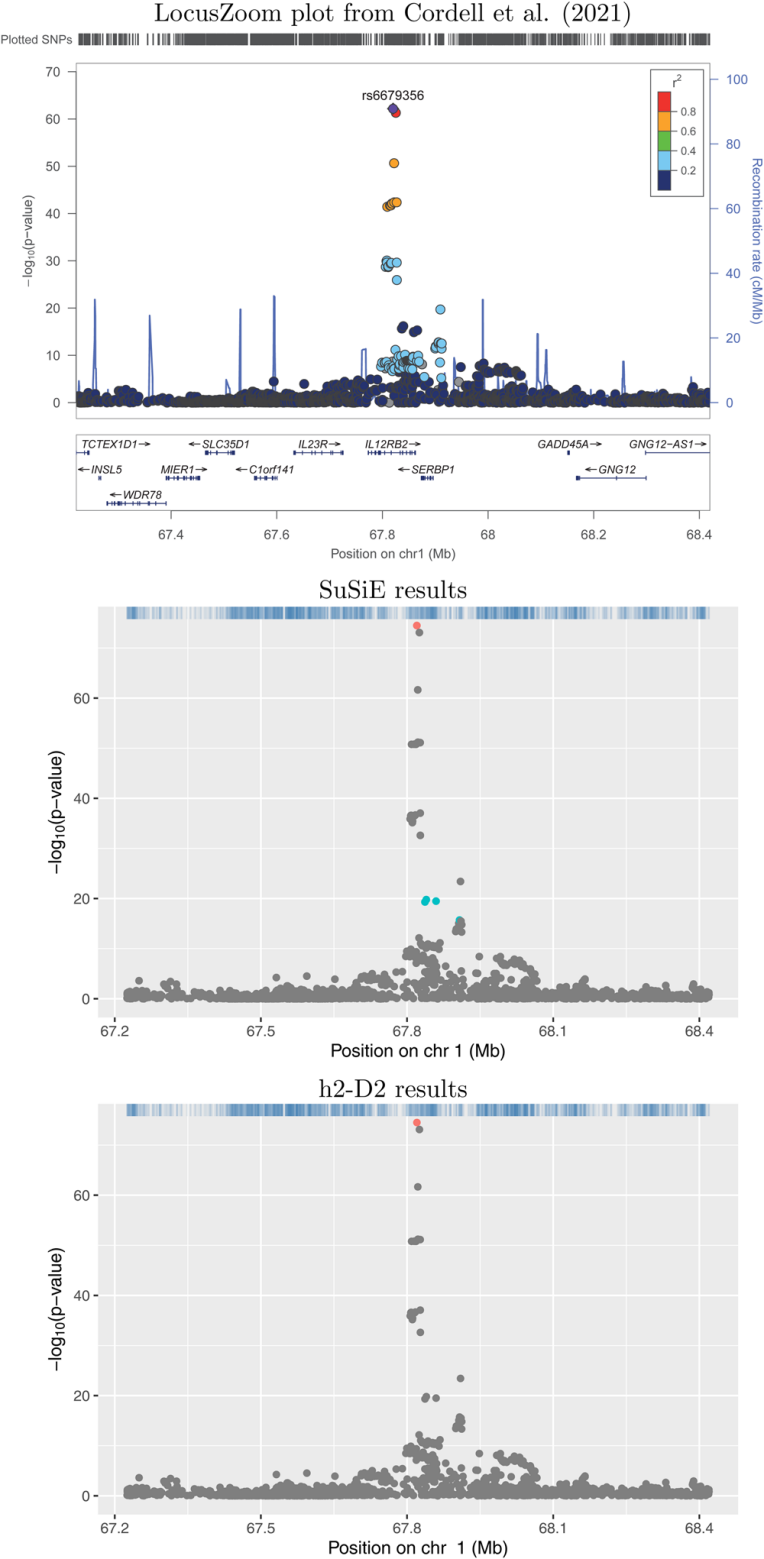
Association plots for 1p31.3. The *x*‐axis shows the base pair position and the *y*‐axis shows the −log10 *p*‐value of each SNP tested for association by Cordell et al. (2021) (top plot) or by using logistic regression (middle and lower plots); the summary statistics from the logistic regression analyses were used as input to SuSiE‐RSS and h2‐D2. SNPs in different credible sets from SuSiE (middle plot) and h2‐D2 (lower plot) are indicated in different colours. SNPs coloured in grey were not chosen as part of any credible set.

**Figure 4:**
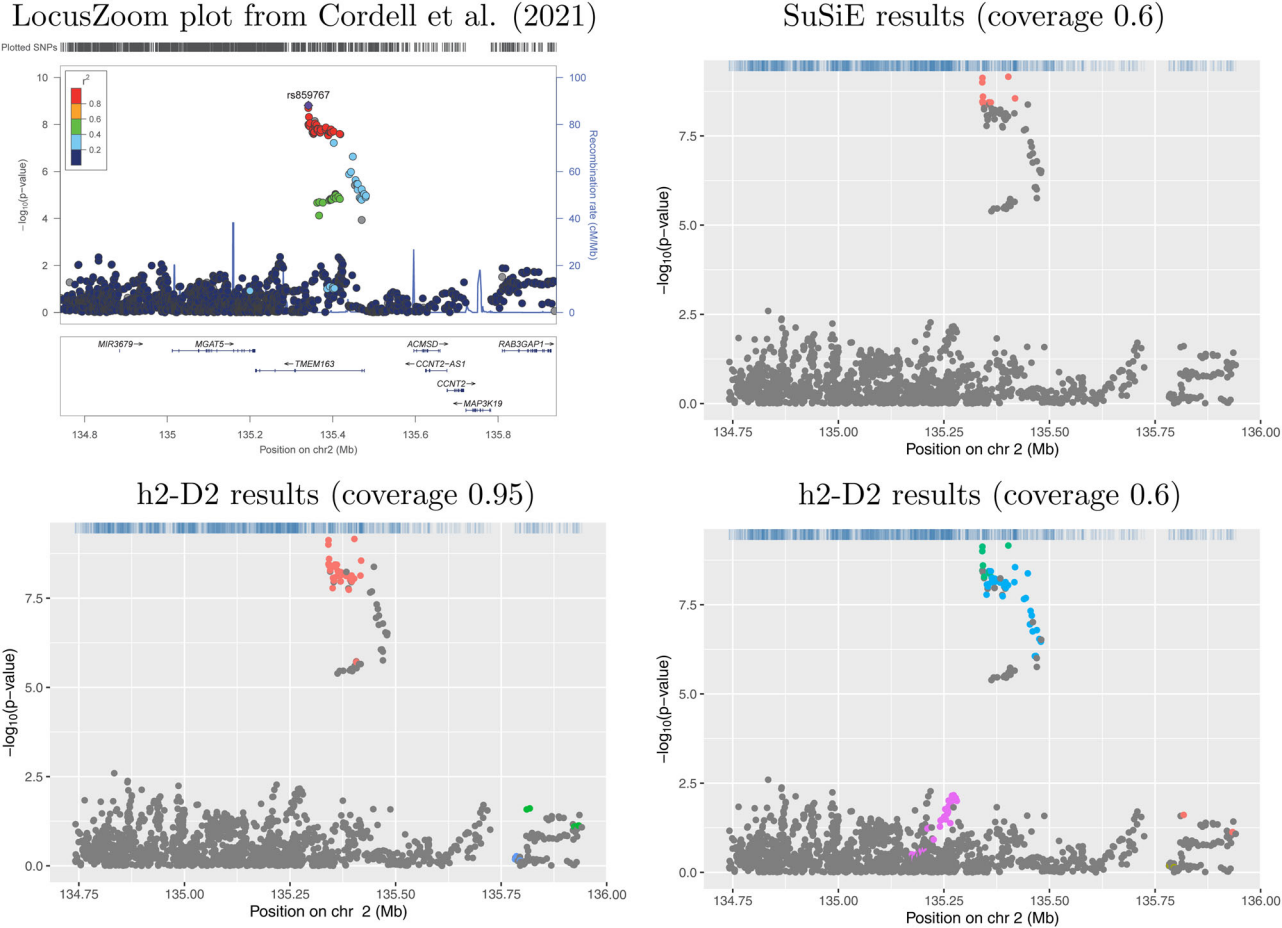
Association plots for 2q21.3. The *x*‐axis shows the base pair position and the *y*‐axis shows the −log10 *p*‐value of each SNP tested for association by Cordell et al. (2021) (top left plot) or by using logistic regression (other plots); the summary statistics from the logistic regression analyses were used as input to SuSiE‐RSS and h2‐D2. SNPs in different credible sets from SuSiE (top right plot) and h2‐D2 (lower plots) are indicated in different colours. SNPs coloured in grey were not chosen as part of any credible set.

Figures 1 and 2 illustrate two loci (13q14.11 and 16p13.13, respectively) where the results from SuSiE‐RSS and h2‐D2 are broadly concordant, identifying either one (Figure 1) or two (Figure 2) credible sets. The main difference seen is that the credible sets from h2‐D2 contain a larger number of variants than those from SuSiE‐RSS.

Figures 3 and 4 illustrate two loci (1p31.3 and 2q21.3, respectively), where the results from SuSiE‐RSS and h2‐D2 are less concordant; in addition to identifying a larger number of SNPs in each credible set, h2‐D2 also identifies different numbers of credible sets than are identified by SuSiE‐RSS (one compared to two from SuSiE‐RSS at 1p31.3, and five compared to one from SuSiE‐RSS at 2q21.3, respectively). At 1p31.3, based on the original LocusZoom plot from Cordell et al. (2021) (Figure 3 top panel), the SuSiE‐RSS results seem most plausible, identifying a second signal at 4 SNPs that are genome‐wide significant but not in strong LD with the primary signal in the region.

The 2q21.3 locus was the one of those where the coverage threshold had to be reduced to get SuSiE‐RSS to generate any credible sets; with this reduced (0.6) threshold, the h2‐D2 results again seem somewhat implausible, potentially over‐estimating the number of credible sets in comparison to SuSiE‐RSS (Figure 4 right‐hand panels) and identifying SNPs that do not look particularly good causal candidates based on their nominal significance levels. If the h2‐D2 coverage threshold is increased back to 0.95, h2‐D2 still identifies two credible sets with implausibly low significance levels (Figure 4 bottom left panel).

In the Supplementary Text, we go through each of the 56 loci in turn, describing the results in detail and arguing (based on the LD pattern in the region shown in the LocusZoom plots of figs. S2.1–S2.57 of Cordell et al. [2021], along with comparison to the previously obtained FINEMAP results), whether the results from SuSiE‐RSS or h2‐D2 seem the most plausible. For the majority of the loci (33 out of 56) we consider the results from SuSiE‐RSS to be most plausible, for 5 loci we consider the results of h2‐D2 to be most plausible, and for 18 of the 56 loci we consider the results from SuSiE‐RSS or h2‐D2 to be equally plausible.

The final full list of implicated variants identified by SuSIE‐RSS (or by h2‐D2 when h2‐D2 was considered more plausible) within each credible set can be found in Table S1.

### 3.2 Initial Simulation Results

We applied SuSiE‐RSS and h2‐D2 to simulated data as described in Section 2. The simulation results are reported in Table 2. Overall the performance of SuSiE‐RSS in these scenarios is better than that of h2‐D2, with SuSiE showing higher power to correctly detect one or both causal variants, and a mean number of identified credible sets (1.77 compared to 1.08) closer to the true number (two) in Scenario 3. We note that the higher power of SuSiE‐RSS compared to h2‐D2 is not fully consistent with results reported by Li, Sham, and Zhang (2024); although in several of scenarios they considered (see fig. 1E of Li, Sham, and Zhang [2024]) SuSiE did have the higher power, in most cases Li, Sham, and Zhang (2024) found h2‐D2 to have higher power than SuSiE. In our study, both methods struggle to identify two credible sets in Scenario 2, probably because of the high LD between the simulated causal variants, meaning that the two signals are generally identified as the presence of a single credible set. The sizes of the credible sets (when generated) are seen to be larger for h2‐D2 than SuSiE‐RSS in Scenarios 1 and 3, again consistent with results reported by Li, Sham, and Zhang (2024).

**Table 2:** Simulation results for different scenarios and metrics.

Analysis		Power	Power	Power	No of CS		CS size	
Method	Scenario	(1st)	(2nd)	(both)	Mean	SD	Mean	SD
SuSiE‐RSS	1	0.95	—	—	0.99	0.10	7.18	2.27
SuSiE‐RSS	2	0.94	0.98	0.92	1.00	0.00	5.27	1.25
SuSiE‐RSS	3	0.85	0.76	0.76	1.77	0.45	7.17	3.15
h2‐D2	1	0.84	—	—	0.90	0.30	9.11	4.76
h2‐D2	2	0.80	0.88	0.72	0.98	0.14	4.18	1.43
h2‐D2	3	0.71	0.23	0.15	1.08	0.46	9.36	5.65

*Note:* Power is the power (probability) to detect the 1st, 2nd or both variants respectively. No of CS is the number of credible sets generated. CS size is the size of the generated credible sets, ignoring the replicates where no credible sets were generated. Mean and SD are the mean and the standard deviation of these quantities over up to 100 simulation replicates.

**Figure 5:**
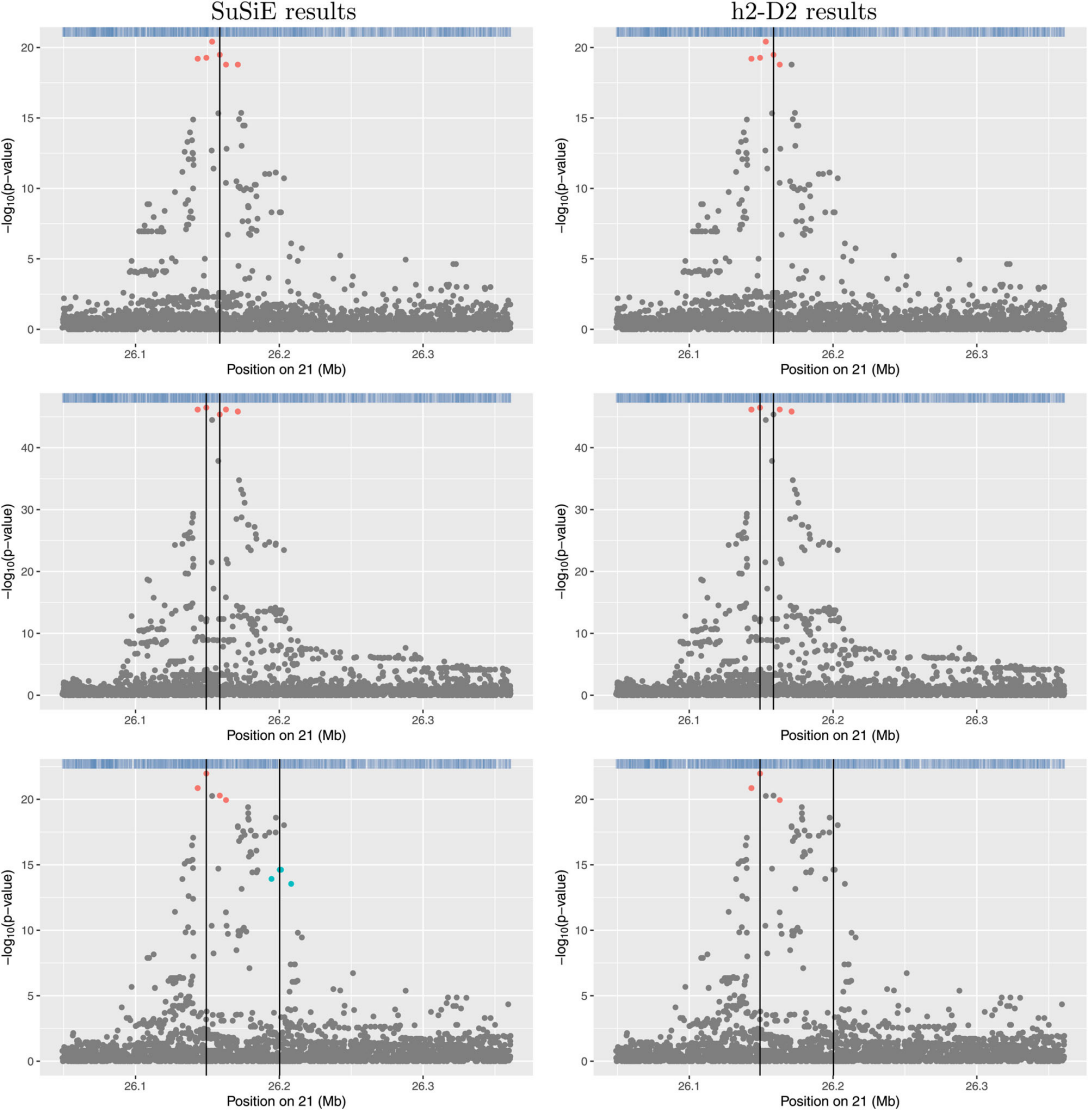
Scatter plots from a single example replicate of simulated data in each of Scenarios 1–3 (top to bottom), with coloured dots highlighting the variants identified and the black vertical line showing the positions of the true disease‐causing variants. The *x*‐axis shows the base pair position and the *y*‐axis shows the –log10 *p*‐value of each SNP tested for association using logistic regression; the summary statistics from the logistic regression analyses were used as input to SuSiE‐RSS and h2‐D2. Results from analysis with SuSiE are shown on the left‐hand plots and results from analysis with h2‐D2 are shown on the right‐hand plots. SNPs coloured in grey were not chosen as part of any credible set.

An illustration of the results in a single replicate of each of the three simulation scenarios is shown in Figure 5. This displays a scatter plot of the negative log‐transformed p‐value in the specified region, with vertical black lines highlighting the positions of the causal SNPs and coloured dots showing the variants that SusiE‐RSS and h2‐D2 have detected in their respective credible set(s). Different colours on the plot correspond to distinct credible sets. In Scenario 1 (top panels), both methods identify a single credible set that includes the simulated causal SNP, along with several other SNPs in LD with it. In Scenario 2 (middle panels), both methods identify a single credible set rather than separate credible sets for each of the two causal SNPs; the credible set from SuSiE‐RSS includes both causal SNPs but that from h2‐D2 only includes one of the two causal SNPs. In Scenario 3 (bottom panels), SuSiE correctly identifies two credible sets, each containing the relevant causal SNP, while h2‐D2 only identifies one credible set, corresponding to the stronger causal SNP. These results serve to illustrate in more detail (for these selected replicates) the overall better performance that we saw with SuSiE‐RSS over the full set of simulation replicates.

### 3.3 Results from Tailored Simulation Study Based on PBC Data

**Figure 6:**
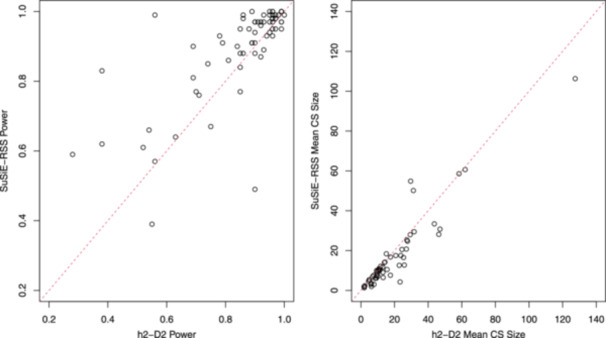
Power (left‐hand plot) and mean credible set (CS) size (right‐hand plot) for SuSiE‐RSS versus h2‐D2. Each dot shows the results from a single simulated causal variant (left‐hand plot) or locus (right‐hand plot), with the power and mean CS calculated over 100 simulation replicates.

The results from our more realistic simulation study are shown in Table 3, the entries of which are summarised in Figure 6 via a comparison of the power to detect each causal variant (left‐hand plot), and the mean size of the credible sets generated at each locus (right‐hand plot). In general, SuSiE‐RSS shows the highest power (more points above the diagonal in the left‐hand plot of Figure 6) and better precision (more points below the diagonal, indicating a small credible set size, in the right‐hand plot), although there are a few loci where h2‐D2 exhibits higher power and/or better precision. Most notably, the size of the credible set generated by h2‐D2 is much smaller than that of SuSiE‐RSS at 17q21.31, similar to what was seen in analysis of the real data. Both methods do reasonably well at detecting, on average, the correct number of credible sets, although the number of credible sets detected by SuSiE‐RSS tends to be less variable, and SuSiE‐RSS also does better at correctly detecting the existence of 4 causal variants (and thus four credible sets) at 3q25.3.

**Table 3:** Results from the tailored simulation study, using the real PBC data (and results) to inform data generation.

			SuSiE results					h2‐D2 results				
Locus			No of CS		CS sizes			No of CS		CS sizes		
number	Locus	Simulated causal variant(s)	Mean	SD	Mean	SD	Power	Mean	SD	Mean	SD	Power
1	1p36.32	rs867436	1.00	0.00	50.14	18.81	0.97	1.74	6.43	31.20	28.83	0.90
2	1p31.3	rs6679356, rs3828069	2.00	0.00	1.97	0.85	0.99, 0.99	2.36	0.50	6.25	8.25	0.56, 0.93
3	1p13.1	rs758518	0.99	0.10	24.71	4.43	0.84	1.03	0.17	27.71	12.58	0.85
4	1q23.1	rs945635	0.99	0.17	10.48	11.02	0.97	1.00	0.14	15.55	15.39	0.99
5	1q31.3	rs12123169	1.00	0.00	8.11	5.07	0.99	1.00	0.00	9.74	6.78	0.96
6	1q32.1	rs55734382	1.00	0.00	16.49	12.07	0.99	1.00	0.00	25.36	16.63	0.98
7	2p25.1	rs891058	0.61	0.49	9.05	5.48	0.61	0.60	0.82	13.05	7.67	0.52
8	2p23.3	rs934613, rs7583409	1.85	0.48	4.54	2.64	0.88, 0.93	1.90	0.30	4.54	2.62	0.90, 0.96
9	2q21.3	rs4953922	0.95	0.22	6.45	6.76	0.95	1.00	0.00	13.20	12.52	0.99
10	2q32.2	rs3771317, rs11889341, rs4343493	3.03	0.17	14.18	10.84	1.00, 0.99, 0.90	3.02	0.20	14.26	10.90	0.96, 1.00, 0.84
11	2q33.2	rs34636506	0.90	0.30	30.77	9.28	0.89	0.95	0.22	47.05	12.37	0.93
12	3p24.3	rs9876137	0.99	0.10	7.61	3.69	0.98	1.08	0.42	17.55	44.17	0.97
13	3p24.2	rs11920829	1.00	0.00	10.66	1.94	0.95	1.02	0.20	11.33	2.92	0.96
14	3q13.33	rs2293370	1.00	0.00	7.16	3.07	0.97	1.02	0.14	9.16	12.37	0.96
15	3q25.33	rs485789, rs77583790, rs582537, rs10513547	4.00	0.14	9.70	12.18	0.99, 0.98, 1.00, 0.99	3.39	0.51	10.38	15.61	0.86, 0.96, 0.89, 0.95
16	4q24(1)	rs6533022	1.00	0.00	60.66	2.80	0.95	1.03	0.22	62.02	12.60	0.88
17	4q24(2)	rs7663401	0.98	0.14	12.71	8.27	0.98	0.97	0.17	25.81	14.65	0.97
18	5p13.2	rs35467801	1.01	0.10	12.10	3.17	0.97	1.00	0.00	11.71	2.99	0.92
19	5q21.1	rs141002831	0.99	0.10	106.30	36.80	0.97	1.03	0.22	127.49	29.88	0.91
20	5q31.3	rs10062349	0.92	0.27	58.58	5.30	0.91	1.05	0.63	58.34	21.23	0.79
21	5q33.3	rs2546890	1.01	0.10	1.30	1.20	0.98	0.99	0.17	1.96	2.44	0.96
22	6q21	rs58926232, rs742108	1.24	0.68	6.16	2.50	0.49, 0.39	1.48	0.61	9.20	3.06	0.90, 0.55
23	6q23.3	rs2327832	1.00	0.00	6.96	0.70	1.00	1.02	0.20	6.92	1.26	0.95
24	6q27	rs10946216	0.69	0.46	28.13	22.31	0.67	0.84	0.55	46.32	27.69	0.75
25	7p21.1	rs13233149	1.00	0.00	17.53	5.51	0.96	1.01	0.10	23.74	25.61	0.92
26	7p14.2‐p14.1	rs60600003	1.00	0.00	29.45	14.00	0.97	1.00	0.00	31.67	14.17	0.95
27	7q32.1	rs34871361, rs3778754	2.47	0.50	11.72	8.11	0.86, 0.91	2.02	0.14	12.95	11.85	0.81, 0.89
28	7q34	rs67134107	0.95	0.22	27.97	15.20	0.77	1.15	0.70	29.30	19.74	0.85
29	8q24.21	rs752429	0.69	0.46	9.87	2.88	0.62	0.39	0.49	11.05	3.30	0.38
30	9q22.33	rs4742711	1.00	0.00	10.85	2.15	0.64	1.00	0.00	10.68	2.30	0.63
31	9q32	rs6478109	0.76	0.43	7.04	2.27	0.76	0.71	0.46	10.03	5.27	0.71
32	10q11.23	rs7097397	0.98	0.14	4.24	6.37	0.95	0.86	0.35	23.29	16.67	0.85
33	11p15.5	rs10398, rs28535720	1.83	0.45	6.04	6.68	0.95, 0.87	1.89	0.31	8.50	11.24	0.97, 0.92
34	11q13.1	rs11601860	1.01	0.10	17.50	7.04	0.97	1.01	0.10	20.73	8.69	0.93
35	11q23.1	rs12419634, rs11213980	1.89	0.31	33.41	23.05	0.94, 0.88	1.92	0.31	43.67	25.94	0.95, 0.86
36	11q23.3	rs11217074	1.00	0.00	10.01	1.95	0.77	1.03	0.30	9.31	3.24	0.70
37	11q24.3	rs10893872	0.67	0.47	3.51	2.97	0.66	0.55	0.50	6.24	4.22	0.54
38	12p13.31	rs1800693	1.01	0.10	1.90	9.05	1.00	1.00	0.14	1.86	8.60	0.99
39	12q24.12	rs10774625	1.00	0.00	3.00	0.00	1.00	1.00	0.14	6.02	30.40	0.99
40	13q14.11	rs141252748	1.02	0.14	20.55	3.58	1.00	1.01	0.17	24.40	5.86	0.96
41	13q14.2‐q14.3	rs9591325	1.00	0.00	5.27	0.86	0.91	0.99	0.10	4.83	1.05	0.89
42	14q13.2	rs712314	0.62	0.49	20.76	3.89	0.59	0.31	0.53	27.03	9.08	0.28
43	14q24.1	rs8008961	1.00	0.00	5.41	1.60	0.88	1.02	0.32	5.15	1.74	0.85
44	14q32.12	rs11624512, rs10137524	1.94	0.31	14.01	6.37	0.94, 0.91	1.96	0.40	13.99	6.42	0.90, 0.90
45	14q32.32	rs59643720	1.00	0.00	3.00	0.00	1.00	1.64	2.51	7.77	20.50	0.99
46	16p13.13	rs12928537, rs243323	2.06	0.24	10.26	5.77	0.98, 0.93	2.02	0.35	10.28	6.45	0.86, 0.94
47	16p12.1	rs1119132	0.98	0.14	9.21	1.30	0.97	0.96	0.20	9.39	1.17	0.90
48	16q22.1	rs79577483	1.00	0.00	54.84	12.20	0.93	2.40	11.31	29.58	38.01	0.78
49	16q24.1	rs11117432	1.00	0.00	2.23	0.47	1.00	1.03	0.30	2.21	0.50	0.99
50	17q12	rs8067378	1.00	0.00	18.38	6.38	0.90	1.47	4.21	15.10	23.86	0.69
51	17q21.31	rs17577094	1.00	0.00	1635.94	454.41	0.83	1.63	2.34	325.06	312.54	0.38
52	18q22.2	rs17207042	1.00	0.00	25.29	2.83	0.99	1.00	0.14	27.31	13.78	0.97
53	19p13.2	rs34725611	1.01	0.10	2.31	3.08	1.00	1.00	0.14	2.31	3.10	0.99
54	19p13.11	rs1811241	0.85	0.36	12.59	8.79	0.85	0.78	0.44	22.79	12.17	0.74
55	19q13.33	rs3745516, rs35228262	1.76	0.43	7.53	4.24	0.97, 0.57	1.68	0.49	7.60	4.39	0.99, 0.56
56	22q13.1	rs137687, rs138384476	1.82	0.41	16.75	14.90	1.00, 0.81	1.72	0.47	17.58	16.44	0.99, 0.69

*Note:* No of CS is the number of credible sets generated. CS size is the size of the generated credible sets, ignoring the replicates where no credible sets were generated. Mean and SD are the mean and the standard deviation of these quantities over up to 100 simulation replicates.

### 3.4 Implicated Genes and Pathways

Identifying the precise number and identity of causal variants in a locus is arguably less important than identifying the biological mechanism through which they operate. Cordell et al. (2021) used the credible sets generated by FINEMAP to identify putative candidate genes via functional annotation of the variants using (among other tools) FUMA (Watanabe et al. 2017). We applied FUMA to the list of implicated variants identified by SuSIE‐RSS (or by h2‐D2 when h2‐D2 was considered more plausible) shown in Table S1. Given the similarity particularly between the SuSiE‐RSS credible sets of putative causal variants and those previously obtained using FINEMAP, it was not surprising that the resulting genes (Table S2) and pathways (Tables S3 and S4) identified were similar to those previously prioritised by Cordell et al. (2021) when using the results obtained from FINEMAP. Consistent with previous work (Cordell et al. 2015), these included pathways such as cytokine signalling, T cell activation, cytokine‐cytokine receptor interaction and the IL12, IL27 and JAK‐STAT signalling pathways. Further biological interrogation/annotation of the putative causal variants listed in Table S1 using alternative software tools and analysis pipelines would be an interesting topic for future work, and could potentially uncover additional biological findings, but is beyond the scope of the current investigation.

## 4 Discussion

Using SuSiE‐RSS and h2‐D2, we successfully identified credible sets of potential causal variants within the 56 established loci (excluding *HLA*) that show strong association with PBC, largely confirming the previous findings obtained using FINEMAP (Cordell et al. 2021). The results from SuSiE‐RSS and h2‐D2 were generally similar, but, where there was a discrepancy, the results from SuSiE‐RSS seemed most plausible in 33 out of the 56 loci interrogated, with h2‐D2 generating the more plausible results at just five of the loci investigated. In computer simulations carried out under a variety of simplistic and more realistic generating scenarios, SuSiE‐RSS also performed better than h2‐D2 in terms of showing higher power to identify true causal variants within the credible set(s), better precision (smaller credible set sizes), and better identification of correct number of credible sets. We note that we used the default parameter options for h2‐D2 in terms of coverage, purity, MCMC iterations, burn‐in, step size etc. (except when tweaking the coverage to match that of SuSiE or to produce any credible sets)—altering these parameters could potentially result in better performance. However, our identification of the higher power of SuSiE‐RSS compared to h2‐D2 is not inconsistent with results reported in several scenarios by Li, Sham, and Zhang (2024), although Li, Sham, and Zhang (2024) did find h2‐D2 to outperform SuSiE (and FINEMAP) in other scenarios. The fact that both we and Li, Sham, and Zhang (2024) find some scenarios where h2‐D2 achieves better performance than SuSiE‐RSS suggests that, in practice, inspection of the results from several complementary analysis approaches may prove fruitful—although the user would then need to make a decision as to which set of results they consider most plausible. The generation of automated approaches for deciding between (or perhaps averaging over) such competing sets of fine‐mapping results would be an interesting topic for future investigation.

We found SuSiE‐RSS to perform better when provided with an in‐sample correlation (LD) matrix compared to using a European reference panel (whose LD may not perfectly match that of the analysed data set), consistent with expected theory. Improving the performance of fine‐mapping methods to better allow for differences between the LD in a reference panel and the target data (when an in‐sample correlation matrix is not available) is an active area of current research. Improvements to allow the inclusion of variants that have not been measured (genotyped or imputed) in all samples would also be extremely useful when analysing summary statistics that derive from meta‐analyses, in which the contributing cohorts may have been genotyped on different genotyping platforms. Such improvements would potentially allow us to combine our European and East Asian PBC cohorts to allow greater fine‐mapping resolution.

## Author Contributions

Heather J. Cordell led the research and designed the project. Aida Gjoka and Heather J. Cordell performed data analysis and Aida Gjoka drafted the manuscript. Both authors contributed to revising the manuscript and approved the final paper.

## Conflicts of Interest

The authors declare no conflicts of interest.

## Supporting information

Variants identified by SuSIE‐RSS within each credible set generated in the PBC analysis

Genes identified by FUMA (SNP2GENE analysis module)

Pathways identified by FUMA (through MAGMA, as implemented within the FUMA SNP2GENE analysis module)

Pathways identified by FUMA (GENE2FUNC analysis module)

Supplementary Information

Supplementary Information

## Data Availability

PBC summary statistics and LD matrices used for the real data analysis can be obtained from https://www.staff.ncl.ac.uk/heather.cordell/GjokaPaper.html. Simulation scripts/simulated data that support the findings of this study are available from the corresponding author upon reasonable request.
