# Supplementary material for: Fine‐Mapping the Results From Genome‐Wide Association Studies of Primary Biliary Cholangitis Using SuSiE and h2‐D2
Source: Genet Epidemiol. 2024 Oct 6;49(1):e22592. doi: 10.1002/gepi.22592 (PMC11656035; doi:10.1002/gepi.22592)
Supplement: Supplementary file 5 — Supplementary Information [file GEPI-49-0-s005.pdf]

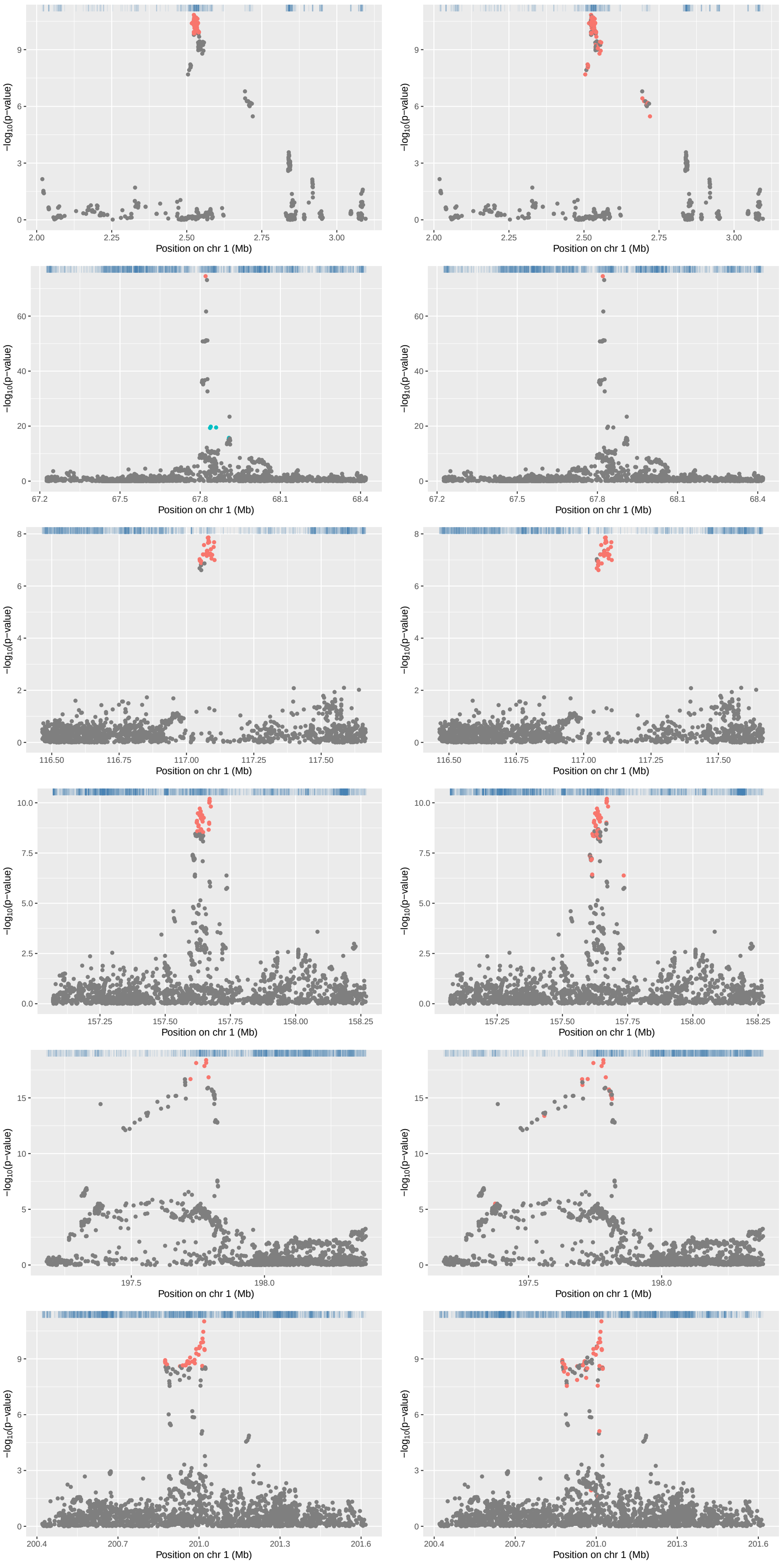

Figure S1: Association plots for six risk loci in chromosome 1 in the order (top–bottom) **1p36.32**, **1p31.3**, **1p13.1**, **1q23.1**, **1q31.3**, **1q32.1**. SNPs colored in grey were not chosen as part of any credible set. SNPs in different credible sets from SuSiE (left-hand plots) and h2-D2 (right-hand plots) are indicated in different colors.

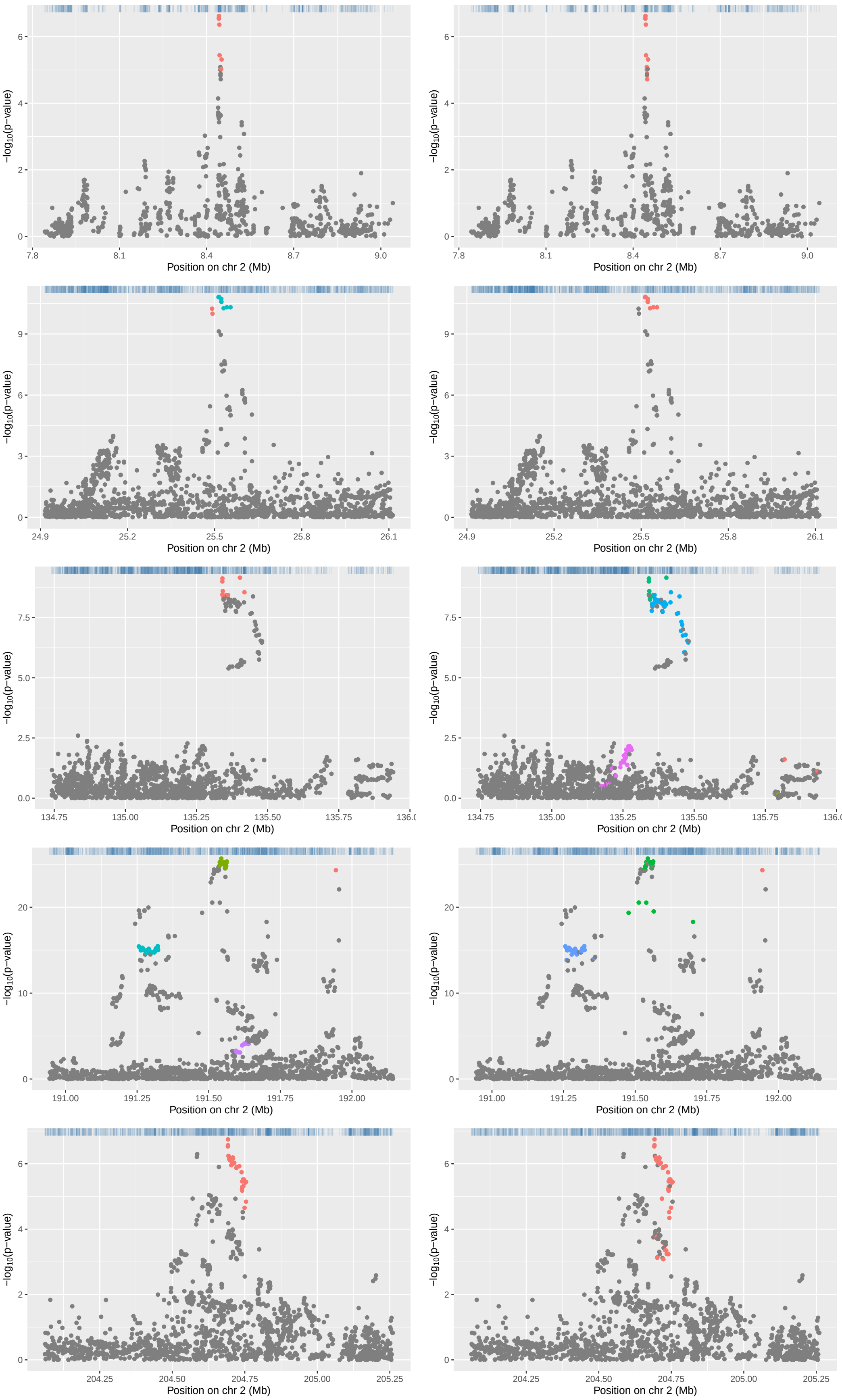

Figure S2: Association plots for five risk loci in chromosome 2 in the order (top–bottom) **2p25.1**, **2p23.3**, **2q21.3**, **2q32.2**, **2q33.2**. SNPs colored in grey were not chosen as part of any credible set. SNPs in different credible sets from SuSiE (left-hand plots) and h2-D2 (right-hand plots) are indicated in different colors.

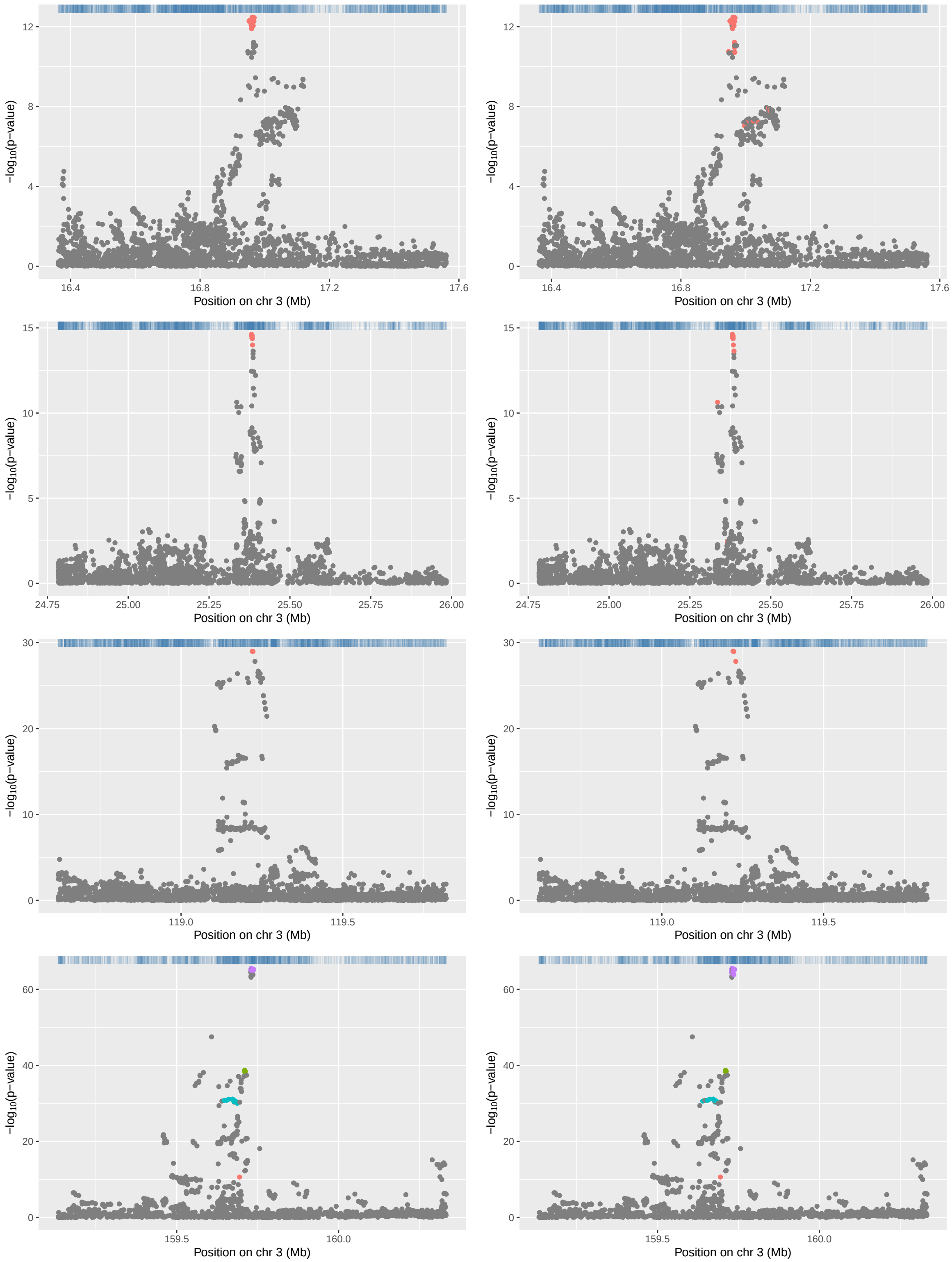

Figure S3: Association plots for four risk loci in chromosome 3 in the order (top–bottom) **3p24.3**, **3p24.2**, **3q13.33**, **3q25.33**. SNPs colored in grey were not chosen as part of any credible set. SNPs in different credible sets from SuSiE (left-hand plots) and h2-D2 (right-hand plots) are indicated in different colors.

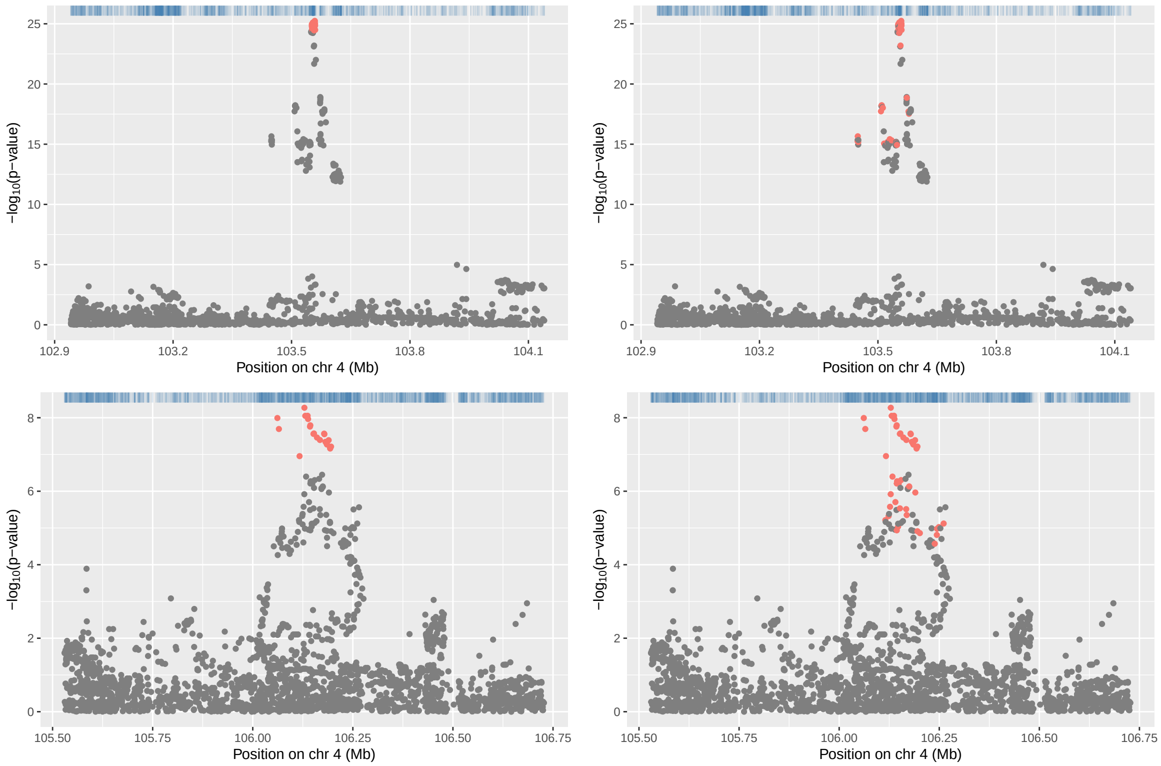

Figure S4: Association plots for two risk loci in chromosome 4 in the order (top–bottom) **4q24(1)**, **4q24(2)**. SNPs colored in grey were not chosen as part of any credible set. SNPs in different credible sets from SuSiE (left-hand plots) and h2-D2 (right-hand plots) are indicated in different colors.

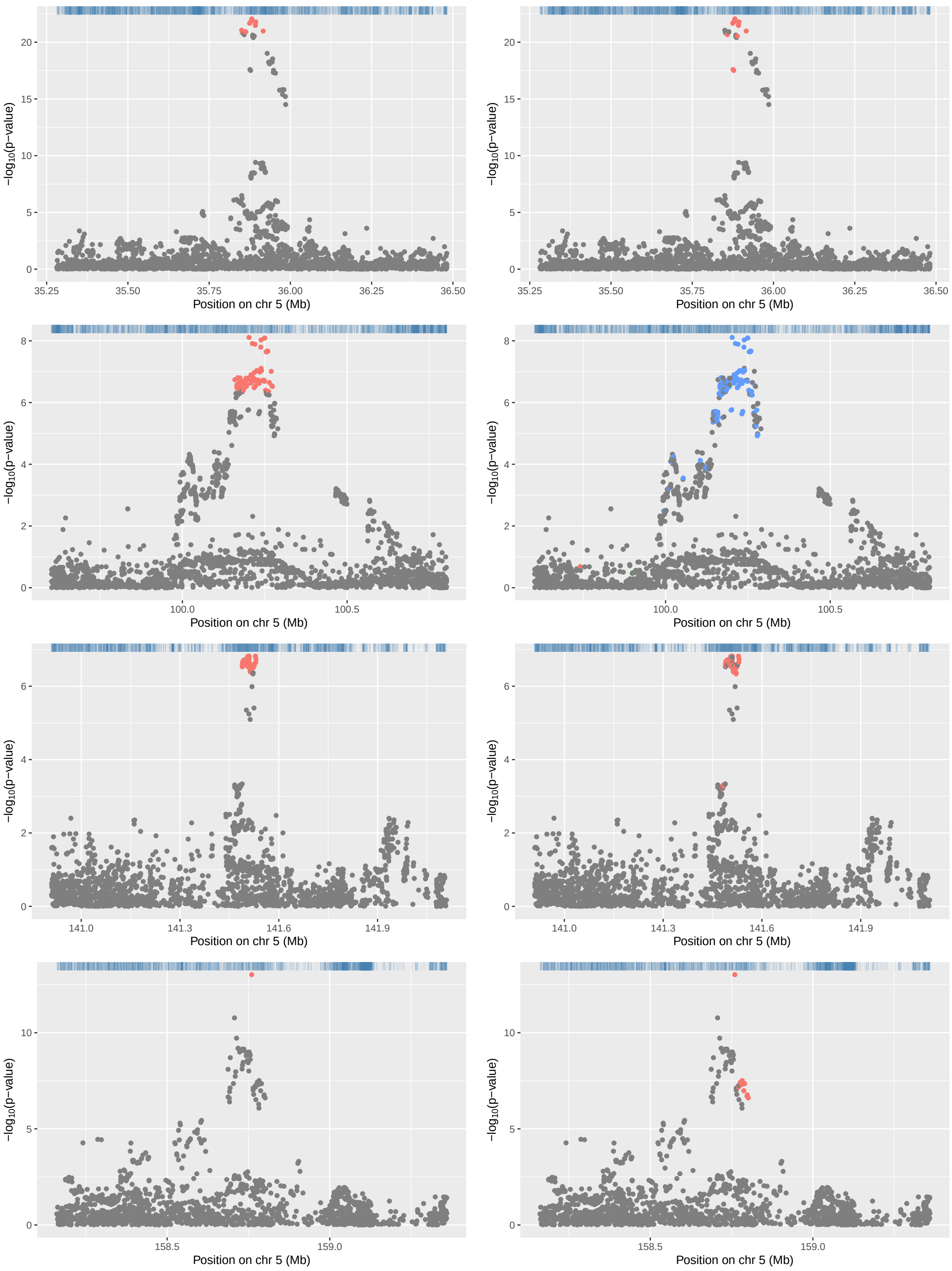

Figure S5: Association plots for four risk loci in chromosome 5 in the order (top–bottom) **5p13.2**, **5q21.1**, **5q31.3**, **5q33.3**. SNPs colored in grey were not chosen as part of any credible set. SNPs in different credible sets from SuSiE (left-hand plots) and h2-D2 (right-hand plots) are indicated in different colors.

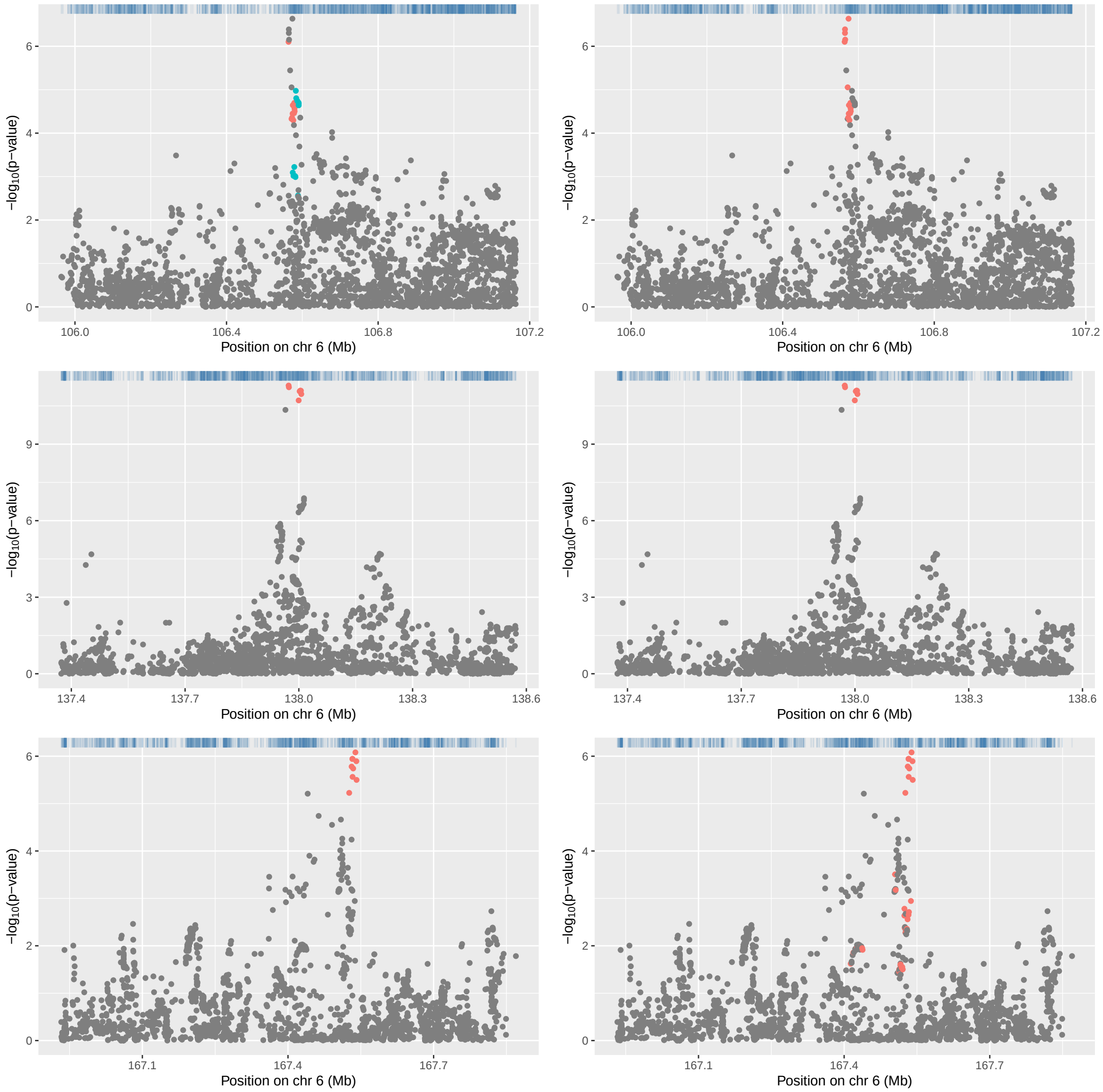

Figure S6: Association plots for three risk loci in chromosome 6 in the order (top–bottom) **6q21**, **6q23.3**, **6q27**. SNPs colored in grey were not chosen as part of any credible set. SNPs in different credible sets from SuSiE (left-hand plots) and h2-D2 (right-hand plots) are indicated in different colors.

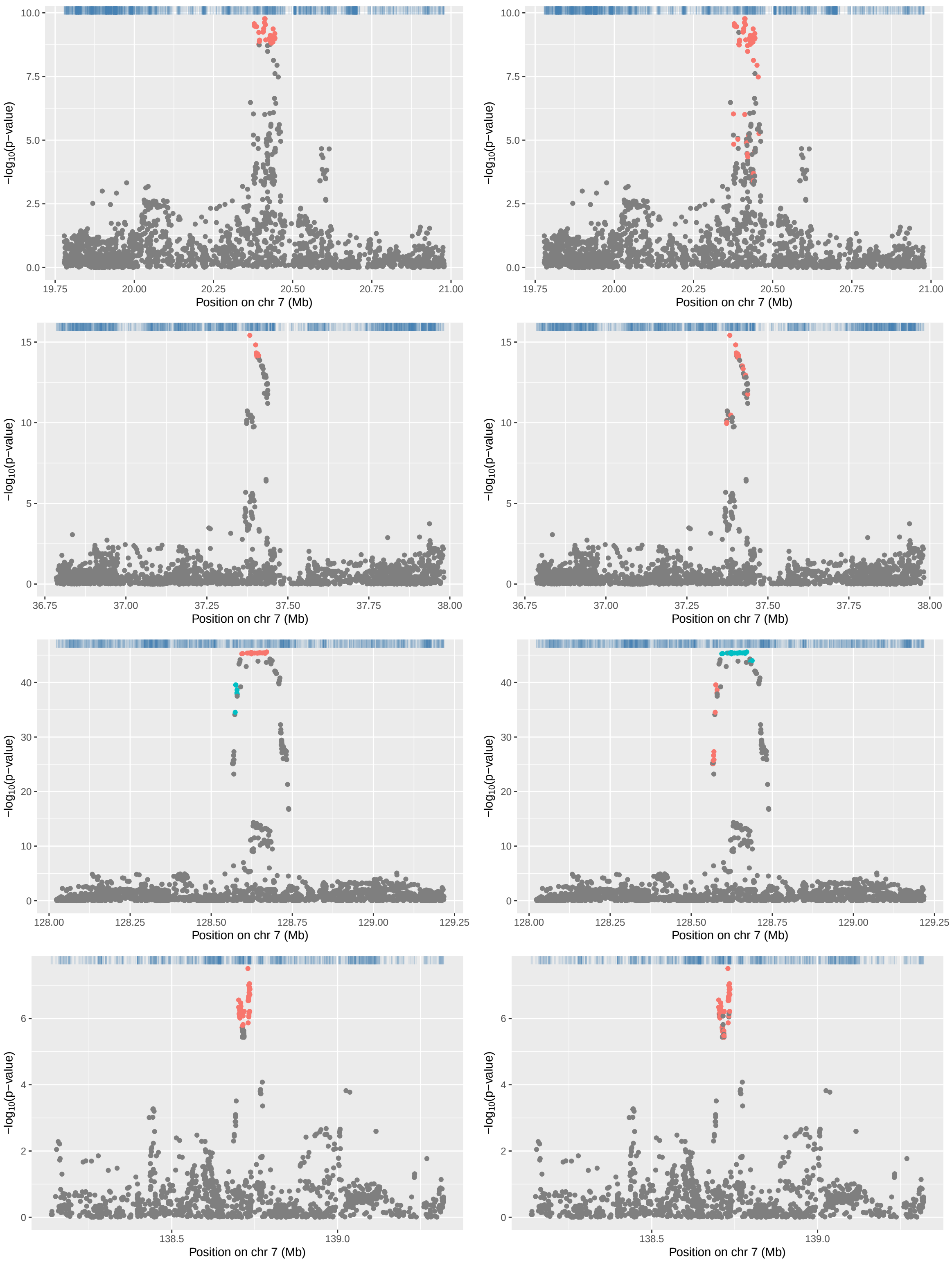

Figure S7: Association plots for four risk loci in chromosome 7 in the order (top–bottom) **7p21.1**, **7p14.2-p14.1**, **7q32.1**, **7q34**. SNPs colored in grey were not chosen as part of any credible set. SNPs in different credible sets from SuSiE (left-hand plots) and h2-D2 (right-hand plots) are indicated in different colors.

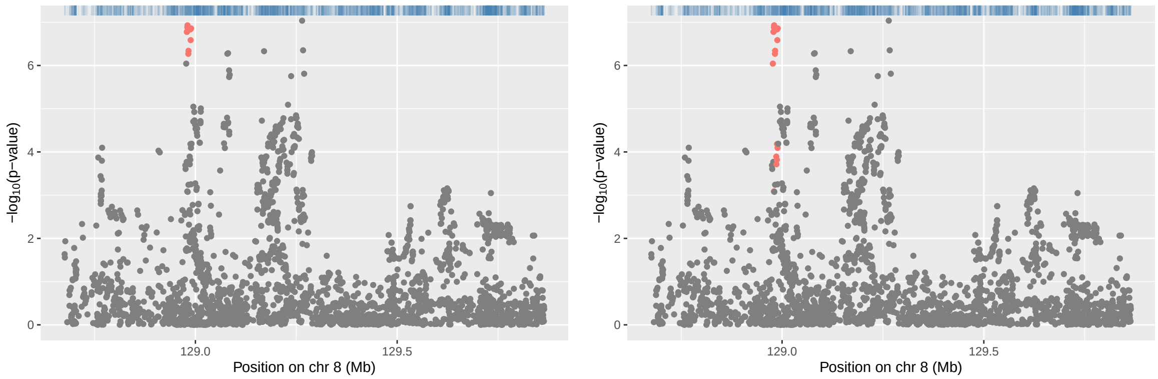

Figure S8: Association plot for one risk locus in chromosome 8, at locus **8q24.21**. SNPs colored in grey were not chosen as part of any credible set. SNPs in different credible sets from SuSiE (left-hand plots) and h2-D2 (right-hand plots) are indicated in different colors.

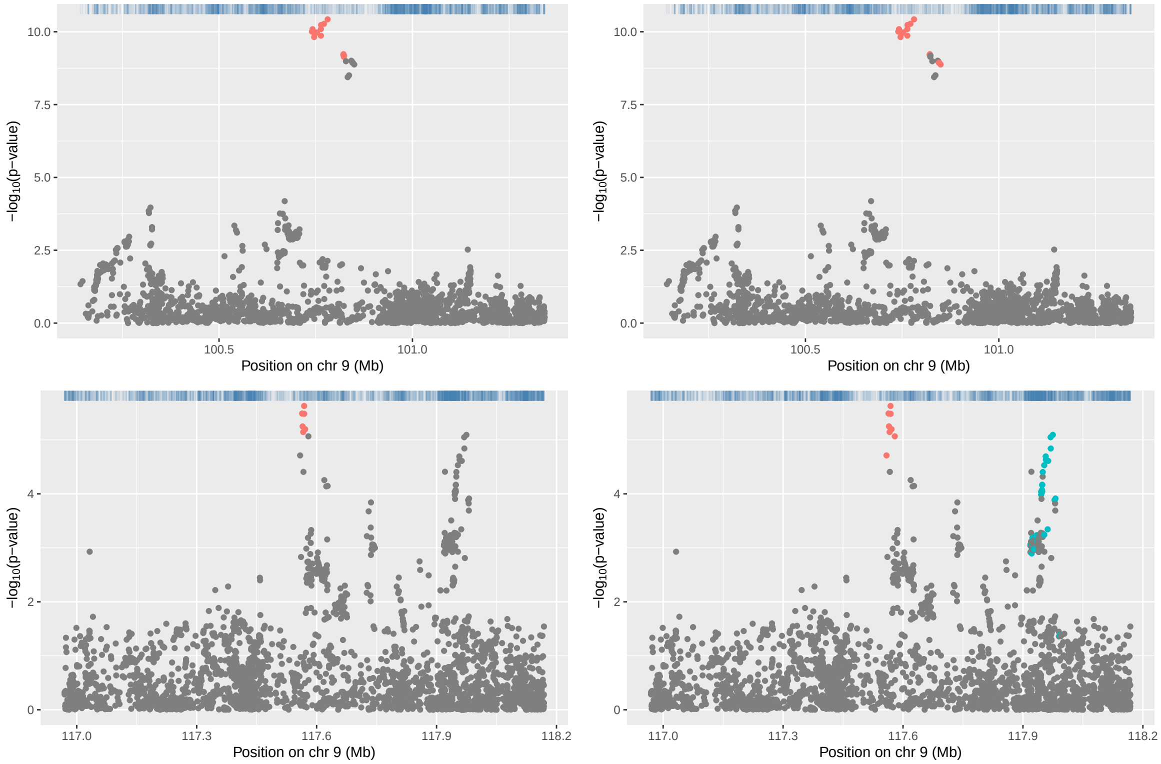

Figure S9: Association plots for two risk loci in chromosome 9 in the order (top–bottom) **9q22.33**, **9q32**. SNPs colored in grey were not chosen as part of any credible set. SNPs in different credible sets from SuSiE (left-hand plots) and h2-D2 (right-hand plots) are indicated in different colors.

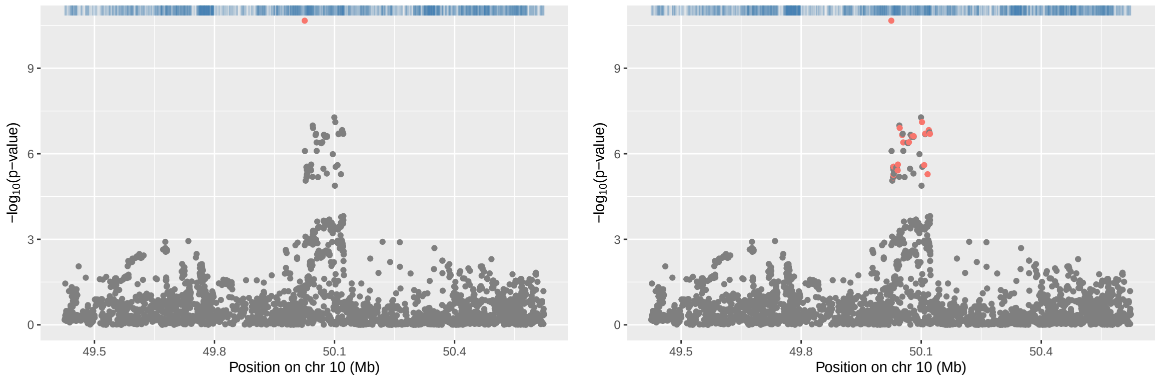

Figure S10: Association plots for one risk locus in chromosome 10, at locus **10q11.23**. SNPs colored in grey were not chosen as part of any credible set. SNPs in different credible sets from SuSiE (left-hand plots) and h2-D2 (right-hand plots) are indicated in different colors.

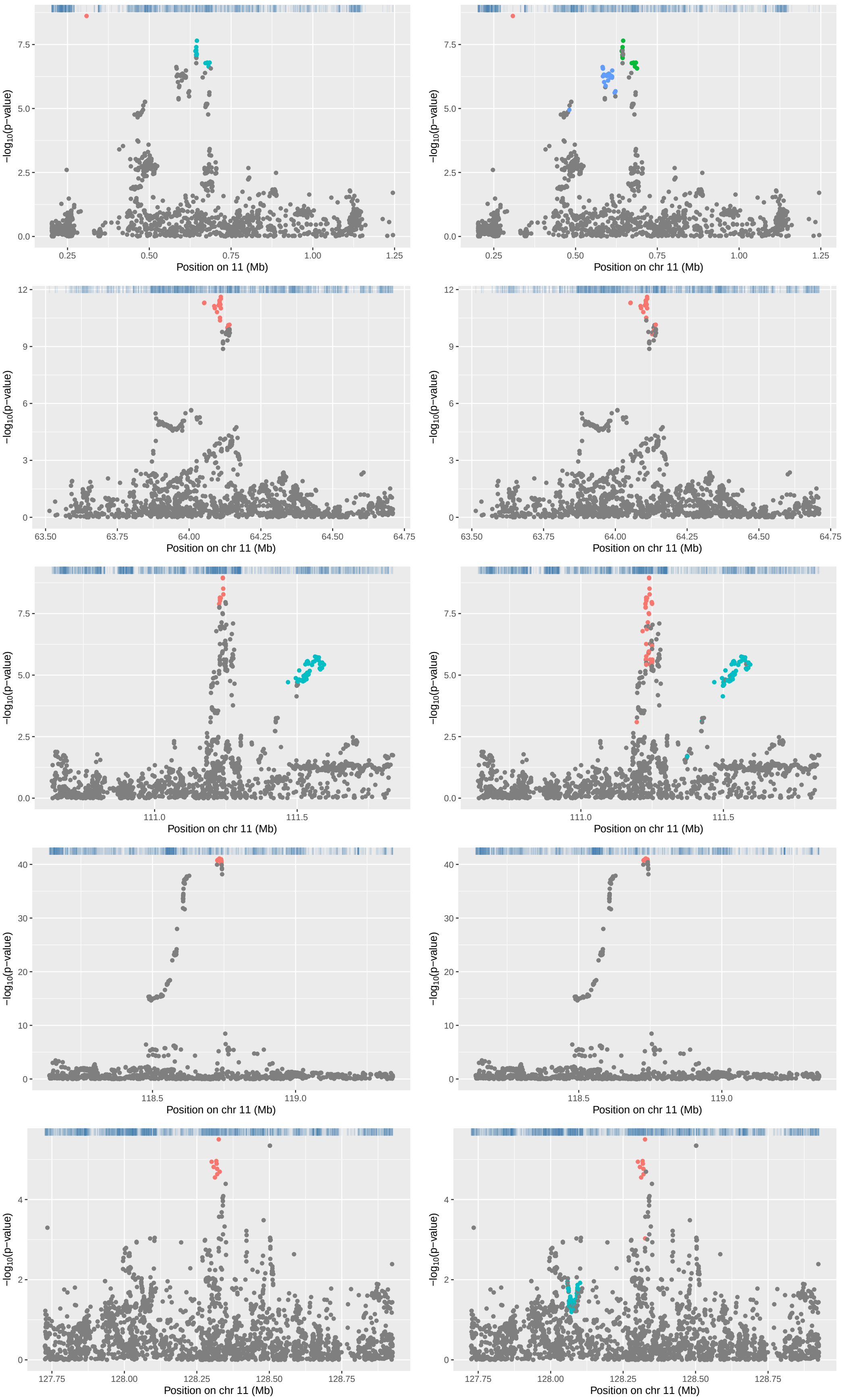

Figure S11: Association plots for five risk loci in chromosome 11 in the order (top–bottom) **11p15.5**, **11q13.1**, **11q23.1**, **11q23.3**, **11q24.3**. SNPs colored in grey were not chosen as part of any credible set. SNPs in different credible sets from SuSiE (left-hand plots) and h2-D2 (right-hand plots) are indicated in different colors.

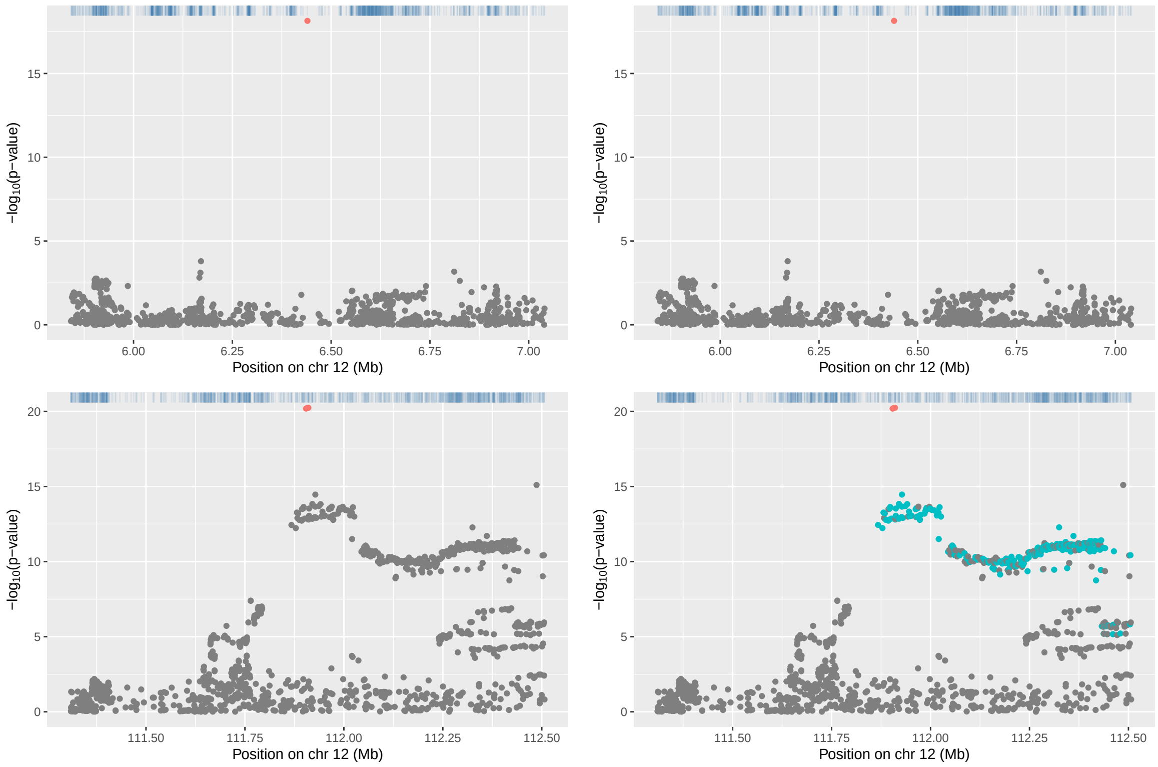

Figure S12: Association plots for two risk loci in chromosome 12 in the order (top–bottom) **12p13.31**, **12q24.12**. SNPs colored in grey were not chosen as part of any credible set. SNPs in different credible sets from SuSiE (left-hand plots) and h2-D2 (right-hand plots) are indicated in different colors.

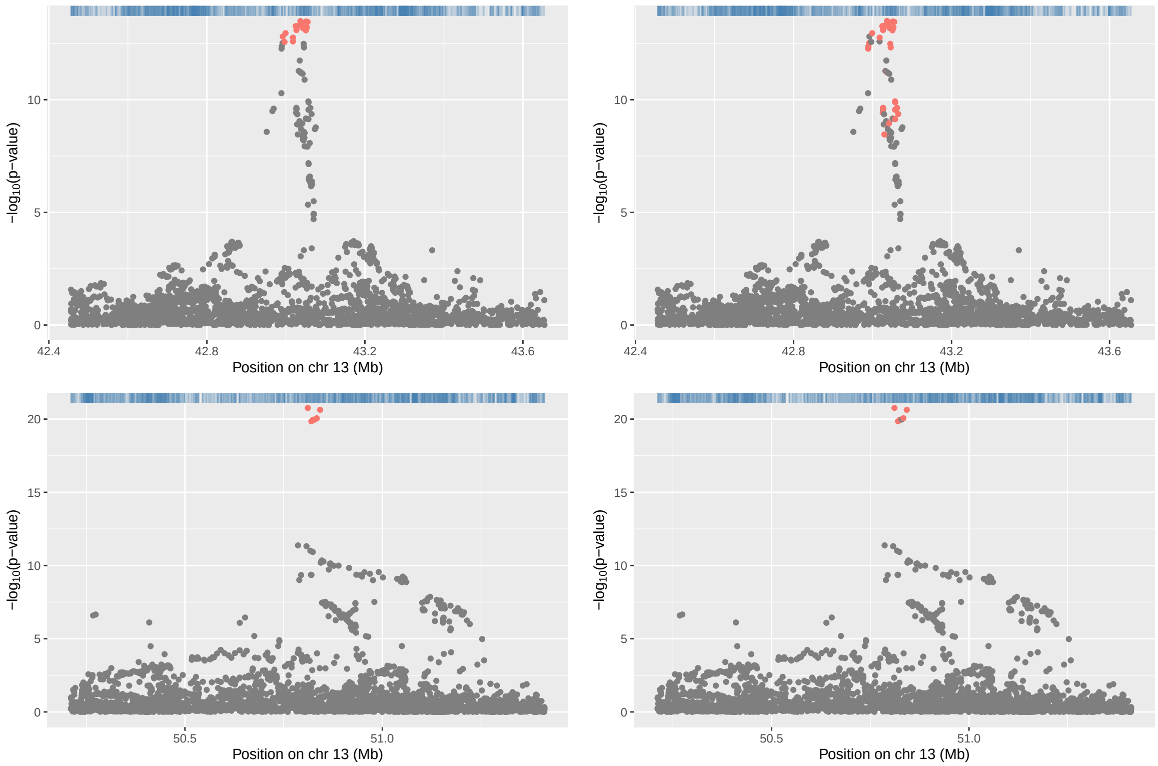

Figure S13: Association plots for two risk loci in chromosome 13 in the order (top–bottom) **13q14.11**, **13q14.2-q14.3**. SNPs colored in grey were not chosen as part of any credible set. SNPs in different credible sets from SuSiE (left-hand plots) and h2-D2 (right-hand plots) are indicated in different colors.

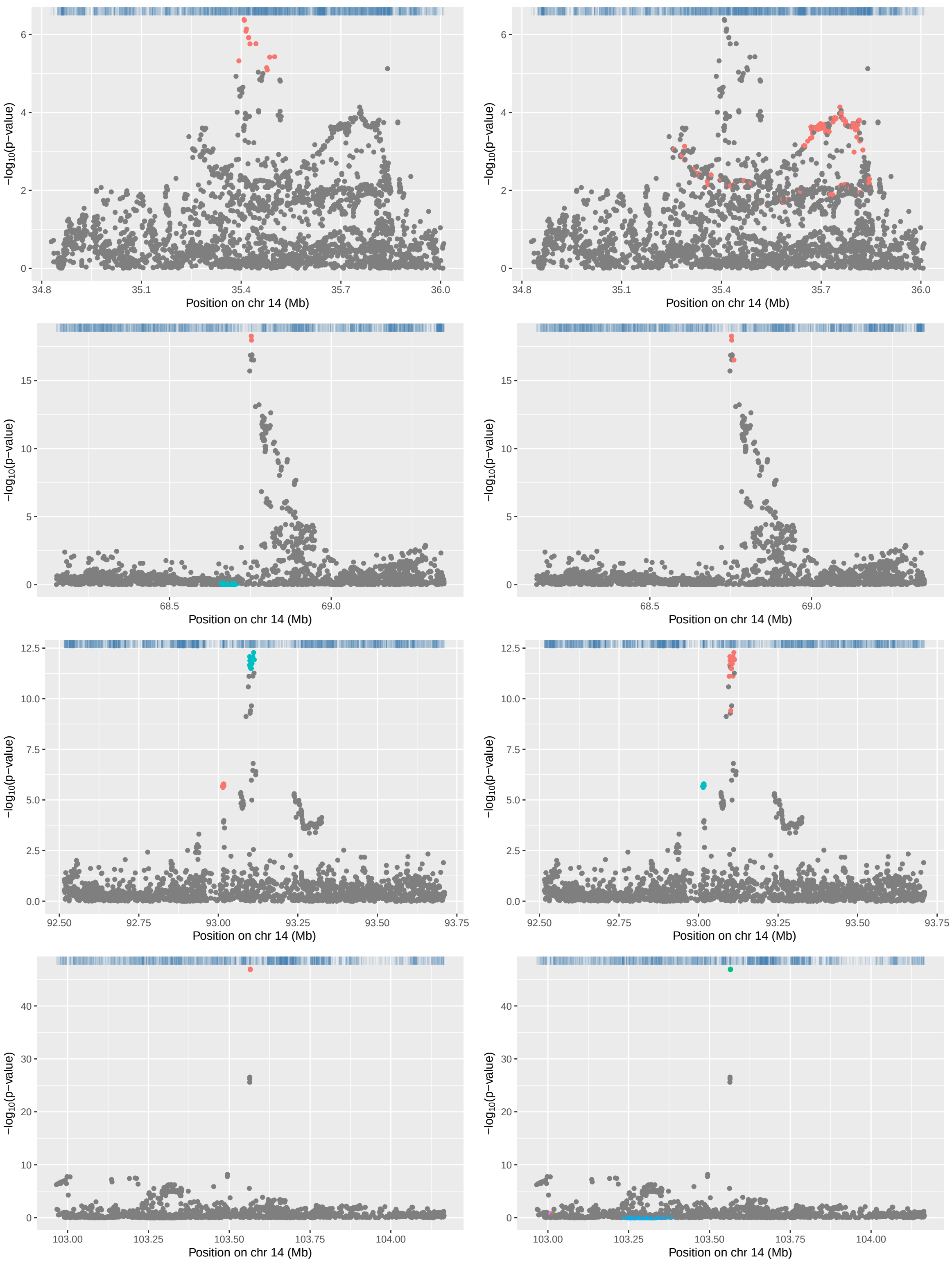

Figure S14: Association plots for four risk loci in chromosome 14 in the order (top–bottom) **14q13.2** and top-right is for **14q24.1**, bottom-left to **14q32.12** and bottom-right to **14q32.32**. SNPs colored in grey were not chosen as part of any credible set. SNPs in different credible sets from SuSiE (left-hand plots) and h2-D2 (right-hand plots) are indicated in different colors.

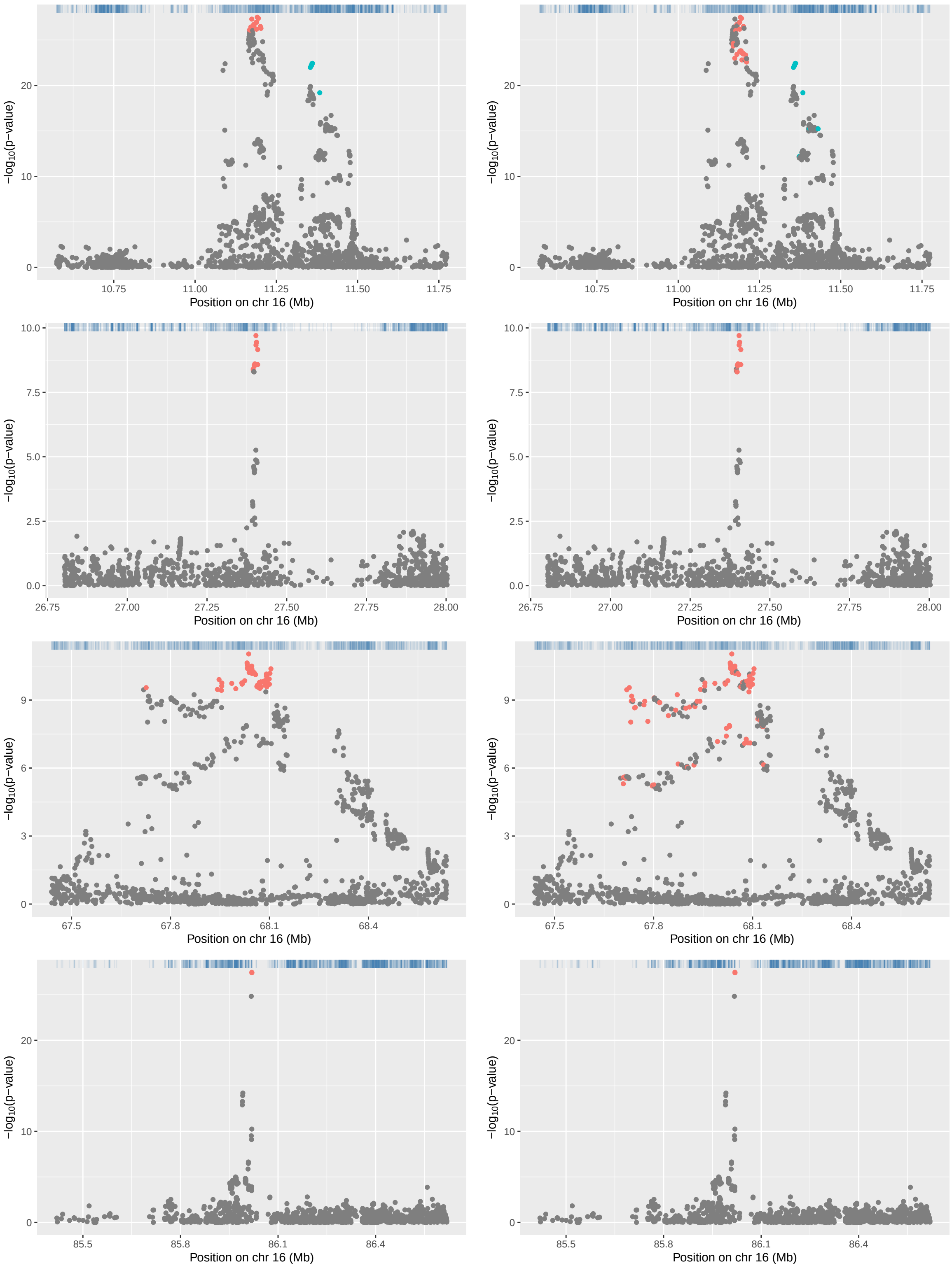

Figure S15: Association plots for four risk loci in chromosome 16 in the order (top–bottom) **16p13.13**, **16p12.1**, **16q22.1**, **16q24.1**. SNPs colored in grey were not chosen as part of any credible set. SNPs in different credible sets from SuSiE (left-hand plots) and h2-D2 (right-hand plots) are indicated in different colors.

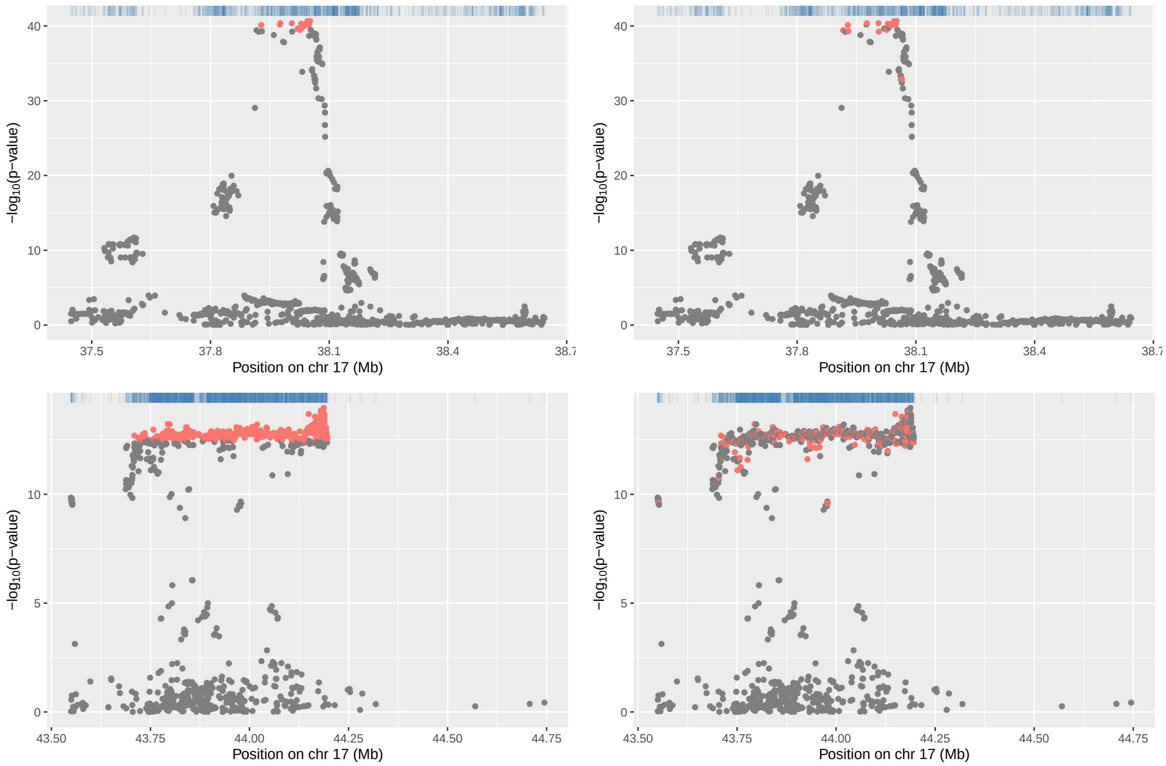

Figure S16: Association plots for two risk loci in chromosome 17 in the order (top–bottom) **17q12**, **17q21.31**. SNPs colored in grey were not chosen as part of any credible set. SNPs in different credible sets from SuSiE (left-hand plots) and h2-D2 (right-hand plots) are indicated in different colors.

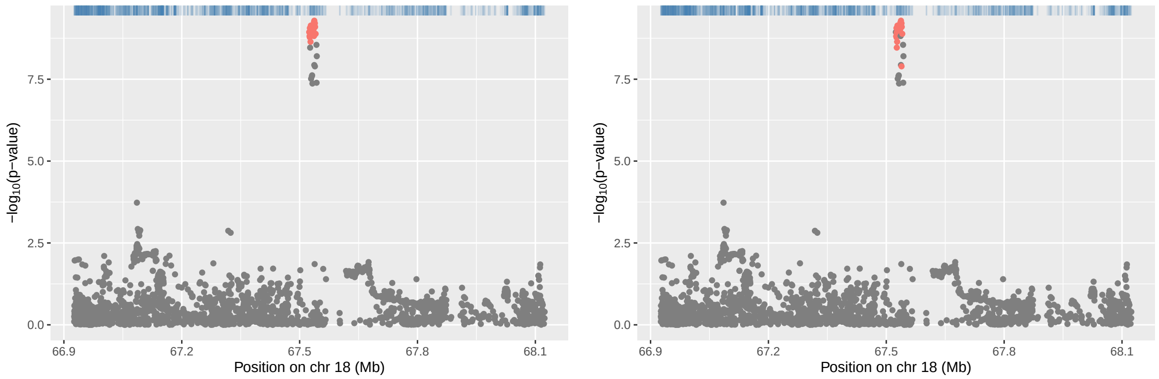

Figure S17: Association plots for one risk locus in chromosome 18, at locus **18q22.2**. SNPs colored in grey were not chosen as part of any credible set. SNPs in different credible sets from SuSiE (left-hand plots) and h2-D2 (right-hand plots) are indicated in different colors.

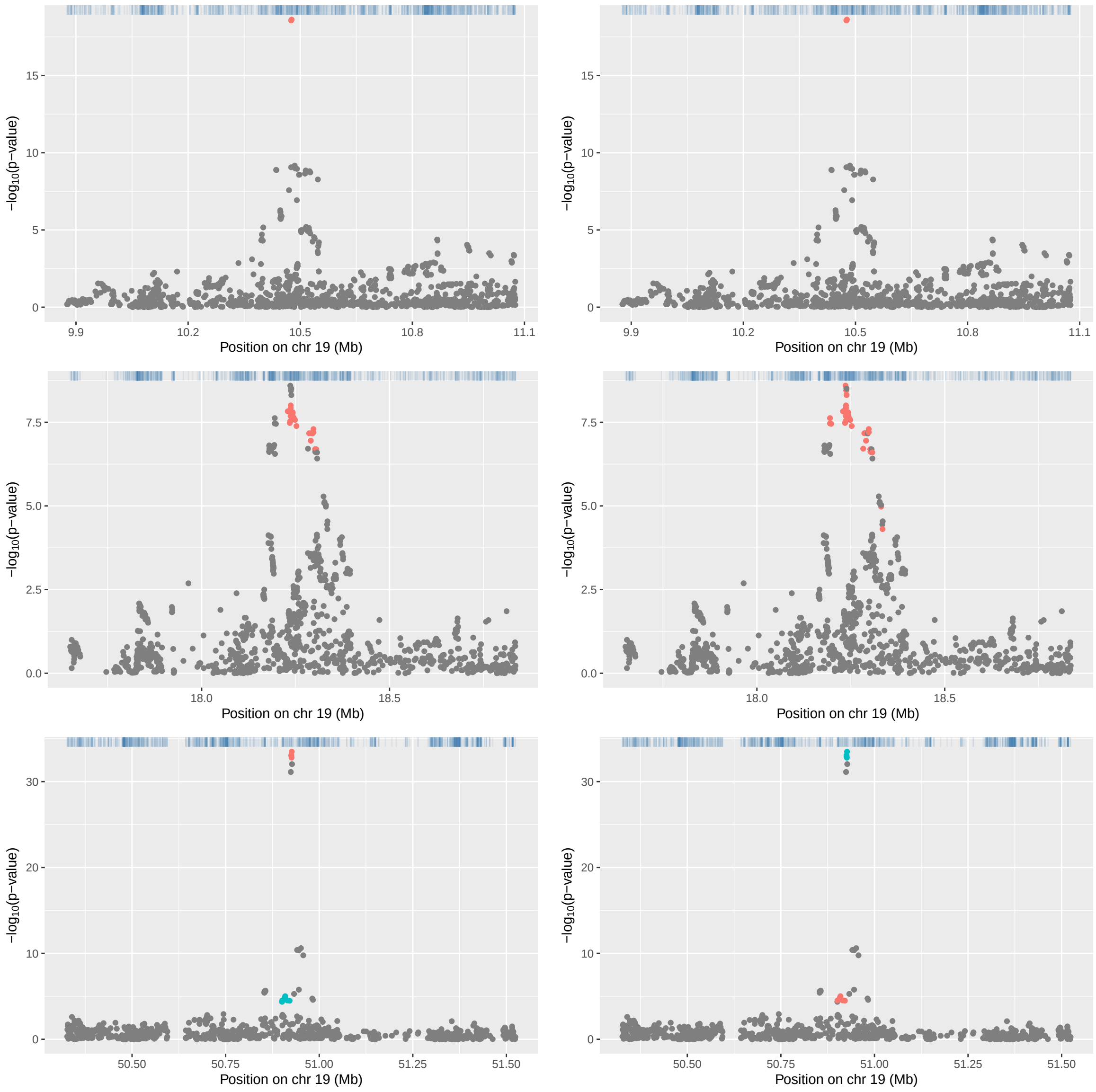

Figure S18: Association plots for three risk loci in chromosome 19 in the order (top–bottom) **19p13.2**, **19p13.11**, **19q13.33**. SNPs colored in grey were not chosen as part of any credible set. SNPs in different credible sets from SuSiE (left-hand plots) and h2-D2 (right-hand plots) are indicated in different colors.

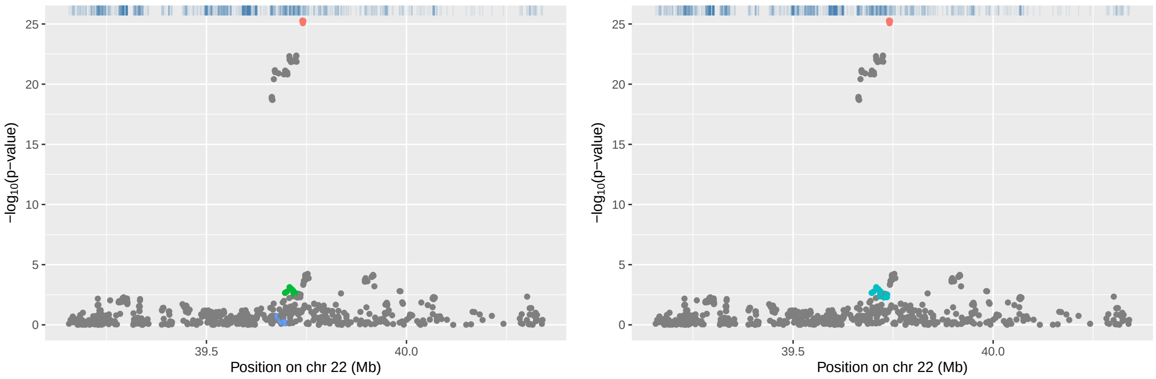

Figure S19: Association plots for one risk locus in chromosome 22, at risk locus **22q13.1**. SNPs colored in grey were not chosen as part of any credible set. SNPs in different credible sets from SuSiE (left-hand plots) and h2-D2 (right-hand plots) are indicated in different colors.
