# Supplementary material for: Fine‐Mapping the Results From Genome‐Wide Association Studies of Primary Biliary Cholangitis Using SuSiE and h2‐D2
Source: Genet Epidemiol. 2024 Oct 6;49(1):e22592. doi: 10.1002/gepi.22592 (PMC11656035; doi:10.1002/gepi.22592)
Supplement: Supplementary file 6 — Supplementary Information [file GEPI-49-0-s006.pdf]

Supplemental Text for
Fine-mapping the results from genome-wide association studies of primary biliary cholangitis using SuSiE and h2-D2

Aida Gjoka and Heather J. Cordell
Population Health Sciences Institute, Newcastle University, UK

Discussion of the 56 PBC-associated loci interrogated

Here we provide more detailed discussion of the results at each of the individual loci presented in Supplementary Figures S1-S19.

Locus 1 (1p36.32)

The top variant from logistic regression is rs867436 at BP position 2523723 (allele T, β=0.1401, SE=0.02075, P=1.448e-11). Both SuSiE-RSS and h2-D2 identify a single credible set, consistent with previous results from FINEMAP. The credible set from SuSiE-RSS is smaller (65 compared to 79 variants) and does not include variants identified by h2-D2 that are seen to be of lower significance than, and at relatively low levels of LD with, the top variant (see Fig S2.1 of Cordell et al. (2021)). For this reason, we consider the results from SuSiE-RSS to be both the most precise and the most compelling.

Locus 2 (1p31.3)

The top variant from logistic regression is rs6679356 at BP position 67820194 (allele C, β=0.4433, SE=0.02415, P=3.112e-75). SuSiE-RSS identifies two credible sets, one set comprising rs6679356 and the other set comprising four variants in LD with one another but not with the top variant (see Fig S2.2 of Cordell et al. (2021)). The top variant in this second signal is rs3828069 at BP position 67839573 (allele C, β=-0.2581, SE=0.02779, P=1.569e-20). h2-D2 identifies only one credible set comprising rs6679356. Given the strong level of significance seen at the second signal, we consider the results from SuSiE-RSS as the most compelling. They are also the most consistent with previous results from FINEMAP, which had suggested either 2 or 3 causal variants at this locus.

Locus 3 (1p13.1)

The top variant from logistic regression is rs758518 at BP position 117081810 (allele G, β=-0.1953, SE=0.03440, P=1.373e-08). Both SuSiE-RSS and h2-D2 identify a single credible set, consistent with previous results from FINEMAP. The size of the credible set is 29 in each case, but slightly different variants are identified by SuSiE-RSS and h2-D2 (with 25 variants found to be in common). Based on

these results, in conjunction with the strong LD pattern in the region (see Fig S2.3 of Cordell et al. (2021)), we consider the results from SuSiE-RSS and h2-D2 at this locus to be equally compelling.

Locus 4 (1q23.1)

The top variant from logistic regression is rs945635 at BP position 157670290 (allele G, β=-0.1299, SE=0.01987, P=6.308e-11). Both SuSiE-RSS and h2-D2 identify a single credible set, consistent with previous results from FINEMAP. The credible set from SuSiE-RSS is smaller (33 compared to 48 variants) and does not include variants identified by h2-D2 that are seen to be of lower significance than, and at lower levels of LD with, the top variant (see Fig S2.4 of Cordell et al. (2021)). For this reason, we consider the results from SuSiE-RSS to be both the most precise and the most compelling.

Locus 5 (1q31.3)

The top variant from logistic regression is rs12123169 at BP position 197780966 (allele A, β=0.2081, SE=0.02329, P=4.042e-19). Both SuSiE-RSS and h2-D2 identify a single credible set, consistent with previous results from FINEMAP. The credible set from SuSiE-RSS is smaller (6 compared to 20 variants) and does not include variants identified by h2-D2 that are seen to be at lower levels of LD with the top variant (see Fig S2.5 of Cordell et al. (2021)). For this reason, we consider the results from SuSiE-RSS to be both the most precise and the most compelling.

Locus 6 (1q32.1)

The top variant from logistic regression is rs55734382 at BP position 201019059 (allele T, β=-0.1469, SE=0.02157, P=9.669e-12). Both SuSiE-RSS and h2-D2 identify a single credible set, consistent with previous results from FINEMAP. The credible set from SuSiE-RSS is smaller (33 compared to 37 variants) and does not include several variants identified by h2-D2 that are seen to be at lower levels of LD with the top variant (see Fig S2.6 of Cordell et al. (2021)). In particular, h2-D2 identifies two variants (rs10920094 and rs35360610) that have considerably lower levels of significance (P=0.01171 and P=0.02620 respectively) than is seen at the top variant. These variants are not identified by SuSiE-RSS. For this reason, we consider the results from SuSiE-RSS to be both the most precise and the most compelling.

Locus 7 (2p25.1)

The top variant from logistic regression is rs891058 at BP position 8442547 (allele A, β=-0.1145, SE=0.02216, P=2.403e-07). Both SuSiE-RSS and h2-D2 identify a single credible set, consistent with previous results from FINEMAP. The credible set from SuSiE-RSS is smaller (7 compared to 11 variants), perhaps reflecting the fact that the coverage threshold for h2-D2 had to be reduced to 0.9 to generate any results. For this reason, we consider the results from SuSiE-RSS to be both the most precise and the most compelling.

Locus 8 (2p23.3)

The top variant from logistic regression is rs934613 at BP position 25514896 (allele T, β=-0.2398, SE=0.03554, P=1.501e-11). SuSiE-RSS identifies two credible sets, one set that includes rs934613, and the other comprising variants rs7583409 (BP position 25491056, allele G, β=0.1378, SE=0.02103,

P=5.752e-11) and rs13428812 (BP position 25492467, allele G, β=0.1354, SE=0.02094, P=1.003e-10), which are not in strong LD with with the top variant (see Fig S2.8 of Cordell et al. (2021)). h2-D2 identifies only one credible set that includes rs934613. Given the high level of significance seen at the rs7583409/rs13428812 signal, we consider the results from SuSiE-RSS as the most compelling. They are also the most consistent with previous results from FINEMAP, which had suggested (with posterior probability 0.8) 2 causal variants at this locus.

Locus 9 (2q21.3)

The top variant from logistic regression is rs4953922 at BP position 135402521 (allele G, β=-0.1697, SE=0.02751, P=6.938e-10) The coverage threshold had to be lowered to 0.6 for SuSiE-RSS to identify a single credible set, consistent with previous results from FINEMAP. With this threshold, h2-D2 identifies five credible sets, including variants that seem to have implausibly weak significance levels (P=0.07433, 0.7108, 0.4545 in sets 1, 2, 5 respectively). If the h2-D2 coverage is increased back to 0.95 (see Figure 4, lower left panel), h2-D2 identifies three credible sets, one (shown in red) that includes rs4953922 and two others (shown in green and blue) that have implausibly weak significance levels (P=0.08085, 0.7108 in sets 2, 3 respectively). Based on these results, we consider the results from SuSiE-RSS to be the most compelling.

Locus 10 (2q32.2)

The top variant from logistic regression is rs3771317 at BP position 191543962 (allele C, β=0.2931, SE=0.02757, P=2.125e-26). SuSiE-RSS identifies four credible sets and h2-D2 identifies three credible sets; both of these are consistent with previous results from FINEMAP, which had suggested (with posterior probability 1.0) 3 causal variants at this locus, when limited to a maximum of 3 for computational reasons. The three credible sets in common between SuSiE-RSS and h2-D2 seem plausible given the LD pattern at, and previous interrogation of, this locus (see Fig S2.10 of Cordell et al. (2021)). The top variants in these three credible sets are rs3771317 at BP position 191543962 (allele C, β=0.2931, SE=0.02757, P=2.125e-26), rs11889341 at BP position 191943742 (allele T, β=0.237, SE=0.02293, P=4.821e-25), and rs4343493 at BP position 191322272 (allele C, β=-0.1778, SE=0.02178, P=3.317e-16), respectively. The fourth credible set, identified only by SuSiE-RSS, seems less plausible given its relatively weak significance level (max P=8.243e-04). Based on these results, we consider the results from h2-D2 to be the most compelling.

Locus 11 (2q33.2)

The top variant from logistic regression is rs34636506 at BP position 204692216 (allele G, β=-0.1055, SE=0.0202, P=1.79e-07) Both SuSiE-RSS and h2-D2 identify a single credible set, consistent with previous results from FINEMAP. The credible set from SuSiE-RSS is smaller (39 compared to 54 variants) and does not include variants identified by h2-D2 that are seen to be of lower significance. For this reason, we consider the results from SuSiE-RSS to be both the most precise and the most compelling.

Locus 12 (3p24.3)

The top variant from logistic regression is rs9876137 at BP position 16961265 (allele G, β=0.1472, SE=0.02023, P=3.341e-13). Both SuSiE-RSS and h2-D2 identify a single credible set, consistent with

previous results from FINEMAP. The credible set from SuSiE-RSS is smaller (14 compared to 25 variants) and does not include variants identified by h2-D2 that are seen to be of lower significance than, and at lower levels of LD with, the top variant (see Fig S2.12 of Cordell et al. (2021)). For this reason, we consider the results from SuSiE-RSS to be both the most precise and the most compelling.

Locus 13 (3p24.2)

The top variant from logistic regression is rs11920829 at BP position 25381124 (allele A, β=0.1582, SE=0.01997, P=2.337e-15). Both SuSiE-RSS and h2-D2 identify a single credible set, consistent with previous results from FINEMAP. The credible set from SuSiE-RSS is smaller (10 compared to 13 variants) and does not include variants identified by h2-D2 that are seen to be of lower significance than, and at lower levels of LD with, the top variant (see Fig S2.13 of Cordell et al. (2021)). For this reason, we consider the results from SuSiE-RSS to be both the most precise and the most compelling.

Locus 14 (3q13.33)

The top variant from logistic regression is rs2293370 at BP position 119219934 (allele A, β=-0.3027, SE=0.02673, P=1.002e-29). Both SuSiE-RSS and h2-D2 identify a single credible set, consistent with previous results from FINEMAP. The credible set from SuSiE-RSS is slightly smaller (2 compared to 3 variants) but both sets look plausible based on the LD pattern in the region (see Fig S2.14 of Cordell et al. (2021)). Based on this, we consider the results from SuSiE-RSS and h2-D2 at this locus to be equally compelling.

Locus 15 (3q25.33)

The top variant from logistic regression is rs485789 at BP position 159730148 (allele T, β=-0.3584, SE=0.02085, P=2.874e-66). Both SuSiE-RSS and h2-D2 identify four credible sets, consistent with previous results from FINEMAP, which had suggested (with posterior probability 1.0) 3 causal variants at this locus, when limited to a maximum of 3 for computational reasons. The sets identified are largely (though not completely) concordent. The top variants in the four credible sets are rs485789 at BP position 159730148 (allele T, β=-0.3584, SE=0.02085, P=2.874e-66), rs77583790 at BP position 159694053 (allele A, β=0.8026, SE=0.1201, P=2.387e-11), rs582537 at BP position 159710098 (allele A, β=0.2639, SE=0.02008, P=1.879e-39), and rs10513547 at BP position 159672597 (allele G, β=0.3050, SE=0.02597, P=7.454e-32), respectively. All identified variants show compelling levels of significance. Based on this, we consider the results from SuSiE-RSS and h2-D2 at this locus to be equally compelling.

Locus 16 (4q24(1))

The top variant from logistic regression is rs6533022 at BP position 103558951 (allele T, β=-0.2099, SE=0.01991, P=5.659e-26). Both SuSiE-RSS and h2-D2 identify a single credible set, consistent with previous results from FINEMAP. The credible set from SuSiE-RSS is smaller (63 compared to 76 variants) and does not include variants identified by h2-D2 that are seen to be of lower significance than, and at lower levels of LD with, the top variant (see Fig S2.16 of Cordell et al. (2021)). For this reason, we consider the results from SuSiE-RSS to be both the most precise and the most compelling.

Locus 17 (4q24(2))

The top variant from logistic regression is rs7663401 at BP position 106128954 (allele C, β=-0.1217, SE=0.02085, P=5.342e-09). Both SuSiE-RSS and h2-D2 identify a single credible set, consistent with previous results from FINEMAP. The credible set from SuSiE-RSS is smaller (23 compared to 48 variants), perhaps reflecting the fact that the coverage threshold for h2-D2 had to be reduced to 0.9 to generate any results. Moreover, the credible set from SuSiE-RSS does not include variants identified by h2-D2 that are seen to be of lower significance than, and at lower levels of LD with, the top variant (see Fig S2.17 of Cordell et al. (2021)). For this reason, we consider the results from SuSiE-RSS to be both the most precise and the most compelling.

Locus 18 (5p13.2)

The top variant from logistic regression is rs35467801 at BP position 35881130 (allele GT, β=-0.2269, SE=0.0231, P=8.796e-23). Both SuSiE-RSS and h2-D2 identify a single credible set, consistent with previous results from FINEMAP. The credible set from SuSiE-RSS is slightly smaller (15 compared to 16 variants) and does not include two variants identified by h2-D2 that are seen to be at lower levels of LD with the top variant (see Fig S2.18 of Cordell et al. (2021)). For this reason, we consider the results from SuSiE-RSS to be both the most precise and the most compelling.

Locus 19 (5q21.1)

The top variant from logistic regression is rs141002831 at BP position 100202282 (allele T, β=0.1196, SE=0.02071, P=7.788e-09). SuSiE-RSS identifies a single credible set of size 87, consistent with previous results from FINEMAP. h2-D2 identifies 3 credible sets of size (1, 1, 126) respectively. The h2-D2 sets of size 1 have implausibly weak significance levels (P=0.2077 at rs72660727 and P=0.3252 at rs72660730). For this reason, we consider the results from SuSiE-RSS to be the most compelling.

Locus 20 (5q31.3)

The top variant from logistic regression is rs10062349 at BP position 141509597 (allele G, β=-0.1082, SE=0.0206, P=1.505e-07). Both SuSiE-RSS and h2-D2 identify a single credible set, consistent with previous results from FINEMAP. The credible set from SuSiE-RSS is slightly smaller (61 compared to 66 variants) and does not include several variants identified by h2-D2 that are both less significant than, and seen to be at lower levels of LD with, the top variant (see Fig S2.20 of Cordell et al. (2021)). For this reason, we consider the results from SuSiE-RSS to be both the most precise and the most compelling.

Locus 21 (5q33.3)

The top variant from logistic regression is rs2546890 at BP position 158759900 (allele G, β=-0.1481, SE=0.01989, P=9.488e-14). Both SuSiE-RSS and h2-D2 identify a single credible set, consistent with previous results from FINEMAP. The credible set from SuSiE-RSS is slightly smaller (1 compared to 10 variants) and does not include several variants identified by h2-D2 that are both less significant than, and seen to be at lower levels of LD with, the top variant (see Fig S2.21 of Cordell et al. (2021)). For this reason, we consider the results from SuSiE-RSS to be both the most precise and the most compelling.

Locus 22 (6q21)

The top variant from logistic regression is rs9486284 at BP position 106573876 (allele C, β=0.1165, SE=0.02253, P=2.318e-07). h2-D2 identifies a single credible set of size 15 containing rs9486284. SuSiE-RSS identifies two credible sets, consistent with previous results from FINEMAP. rs9486284 is not contained in the credible sets output by SuSiE-RSS. Instead, the top SNPs in the two credible sets from SuSiE-RSS are rs58926232 at BP position 106563612 (allele G, β=0.1265, SE=0.02562, P=7.893e-07) and rs742108 at BP position 106582920 (allele A, β=0.117, SE=0.02657, P=1.059e-05) respectively. Comparison of these results with those previously obtained, and perusal of the LD pattern (see Fig S2.23 of Cordell et al. (2021)), suggests that SuSiE-RSS's identification of two separate signals with rs58926232 and rs742108 as the top SNPs is, on balance, most compelling, although the top SNP from h2-D2 (rs9486284) should not be discounted. Further interrogation of this signal is warranted, although this is complicated by the relatively modest level of association seen.

Locus 23 (6q23.3)

The top variant from logistic regression is rs2327832 at BP position 137973068 (allele G, β=0.1600, SE=0.02317, P=5.037e-12). Both SuSiE-RSS and h2-D2 identify a single credible set containing the same 7 variants, which is inconsistent with previous results from FINEMAP (which had rather suggested 2 or 3 causal variants at this locus, perhaps picking up the more modest signals seen on either side of the top signal). We consider the results from SuSiE-RSS and h2-D2 at this locus to be equally compelling.

Locus 24 (6q27)

The top variant from logistic regression is rs10946216 at BP position 167538897 (allele T, β=0.09847, SE=0.01998, P=8.301e-07). Both SuSiE-RSS and h2-D2 identify a single credible set, consistent with previous results from FINEMAP. The credible set from SuSiE-RSS is smaller (10 compared to 44 variants), perhaps reflecting the fact that the coverage threshold for h2-D2 had to be reduced to 0.9 to generate any results. The credible set from SuSiE-RSS does not include variants identified by h2-D2 that are seen to be of lower significance than, and at lower levels of LD with, the top variant (see Fig S2.25 of Cordell et al. (2021)). For this reason, we consider the results from SuSiE-RSS to be both the most precise and the most compelling.

Locus 25 (7p21.1)

The top variant from logistic regression is rs13233149 at BP position 20411514 (allele C, β=0.1343, SE=0.02103, P=1.712e-10). Both SuSiE-RSS and h2-D2 identify a single credible set, consistent with previous results from FINEMAP. The credible set from SuSiE-RSS is smaller (27 compared to 48 variants) and does not include variants identified by h2-D2 that are seen to be of lower significance than, and at lower levels of LD with, the top variant (see Fig S2.26 of Cordell et al. (2021)). For this reason, we consider the results from SuSiE-RSS to be both the most precise and the most compelling.

Locus 26 (7p14.2-p14.1)

The top variant from logistic regression is rs60600003 at BP position 37382465 (allele G, β=0.2605, SE=0.03198, P=3.835e-16). Both SuSiE-RSS and h2-D2 identify a single credible set, consistent with previous results from FINEMAP. The credible set from SuSiE-RSS is smaller (33 compared to 44 variants) and does not include variants identified by h2-D2 that are seen to be of lower significance

than, and at lower levels of LD with, the top variant (see Fig S2.27 of Cordell et al. (2021)). For this reason, we consider the results from SuSiE-RSS to be both the most precise and the most compelling.

Locus 27 (7q32.1)

The top variant from logistic regression is rs34871361 at BP position 128671086 (allele T, β=0.4136, SE=0.02894, P=2.501e-46). Consistent with FINEMAP, both SuSiE-RSS and h2-D2 identify two credible sets, one that includes rs34871361 and one whose top SNP is rs3778754 at BP position 128575552 (allele G, β=0.2672 SE=0.02011, P=2.693e-40). The credible sets from SuSiE-RSS are smaller in size and do not include variants identified by h2-D2 that are seen to be of lower significance than, and at lower levels of LD with, the top variants (see Fig S2.28 of Cordell et al. (2021)). For this reason, we consider the results from SuSiE-RSS to be both the most precise and the most compelling.

Locus 28 (7q34)

The top variant from logistic regression is rs67134107 at BP position 138729543 (allele GAAT, β=0.1114, SE=0.02012, P=3.095e-08). Both SuSiE-RSS and h2-D2 identify a single credible set, of similar size, consistent with previous results from FINEMAP. Based on these results, we consider the results from SuSiE-RSS and h2-D2 to be equally compelling.

Locus 29 (8q24.21)

The top variant from logistic regression is rs4733851 at BP position 129264420 (allele A, β=0.1082, SE=0.02026, P=9.189e-08). Both SuSiE-RSS and h2-D2 identify a single credible set, which is inconsistent with previous results from FINEMAP (which had rather suggested 2 causal variants at this locus). Moreover the top SNP identified is not rs4733851 but rather rs752429 at BP position 128980363 (allele T, β=-0.1056, SE=0.01994, P=1.176e-07). The credible set from SuSiE-RSS is smaller (9 compared to 16 variants), perhaps reflecting the fact that the coverage threshold for h2-D2 had to be reduced to 0.9 to generate any results. The credible set from SuSiE-RSS does not include variants identified by h2-D2 that are seen to be of lower significance than, and at lower levels of LD with, the top variant identified (see Fig S2.30 of Cordell et al. (2021)). For this reason, we consider the results from SuSiE-RSS to be both the most precise and the most compelling. However neither SuSiE-RSS or h2-D2 are fully compelling on account of missing rs4733851. Further interrogation of this signal is warranted, although this is complicated by the relatively modest level of association seen.

Locus 30 (9q22.33)

The top variant from logistic regression is rs4742711 at BP position 100780790 (allele A, β=-0.167, SE=0.02525, P=3.743e-11). Both SuSiE-RSS and h2-D2 identify a single credible set, of similar size, consistent with previous results from FINEMAP. Based on these results, we consider the results from SuSiE-RSS and h2-D2 to be equally compelling.

Locus 31 (9q32)

The top variant from logistic regression is rs6478109 at BP position 117568766 (allele A, β=-0.0999, SE=0.02116, P=2.355e-06). SuSiE-RSS identifies a single credible set that includes rs6478109, while h2-D2 identifies two credible sets, one that includes rs6478109 and one for which the top SNP is rs10817726

at BP position 117974717 (allele T, β=0.13900, SE=0.03115, P=8.072e-06). These sets were obtained using a coverage threshold of 0.7, as required by both SuSiE-RSS and h2-D2 to generate any results. These results are both consistent with FINEMAP which had suggested 1 or 2 causal variants with posterior probabilities 0.55 and 0.44 respectively. Based on these (relatively weak) association results and the LD pattern shown in Fig S2.32 of Cordell et al. (2021), we consider the results from SuSiE-RSS and h2-D2 to be equally compelling. We note that this region is unusual in showing a much stronger association signal in the Asian rather than the European cohorts (Cordell et al., 2021).

Locus 32 (10q11.23)

The top variant from logistic regression is rs7097397 at BP position 50025396 (allele A, β=-0.1395, SE=0.02084, P=2.153e-11). Both SuSiE-RSS and h2-D2 identify a single credible set, consistent with previous results from FINEMAP. The credible set from SuSiE-RSS is smaller (1 compared to 19 variants) and does not include variants identified by h2-D2 that are seen to be of lower significance than, and at lower levels of LD with, the top variant (see Fig S2.33 of Cordell et al. (2021)). For this reason, we consider the results from SuSiE-RSS to be both the most precise and the most compelling.

Locus 33 (11p15.5)

The top variant from logistic regression is rs10398 at BP position 308180 (allele G, β=-0.1539, SE=0.02580, P=2.423e-09). This variant does not correspond to the signal identified by Cordell et al. (2021); the coverage threshold had to be lowered to 0.7 for SuSiE-RSS to identify the signal from Cordell et al. (2021). With this threshold, consistent with FINEMAP, SuSiE-RSS identifies two credible sets, one that includes rs10398 and the other whose top SNP is rs28535720 at BP position 645569 (allele C, β=-0.1129, SE=0.02019, P=2.241e-08). With this same threshold, h2-D2 identifies three credible sets of which the first two correspond to those from SuSiE-RSS and the third is of slightly lower significance. On balance, based on the better precision from SuSiE-RSS and the better consistency with FINEMAP, we consider the results from SuSiE-RSS to be both the most precise and the most compelling.

Locus 34 (11q13.1)

The top variant from logistic regression is rs11601860 at BP position 64110422 (allele T, β=-0.1458, SE=0.0208, P=2.411e-12). Both SuSiE-RSS and h2-D2 identify a single credible set, of identical size, consistent with previous results from FINEMAP. Based on these results, we consider the results from SuSiE-RSS and h2-D2 to be equally compelling.

Locus 35 (11q23.1)

The top variant from logistic regression is rs12419634 at BP position 111239365 (allele G, β=-0.1259, SE=0.02067, P=1.10e-09). Consistent with FINEMAP, both SuSiE-RSS and h2-D2 identify two credible sets, one that includes rs12419634 and one whose top SNP is rs11213980 at BP position 111562692 (allele A, β=0.1045 SE=0.02186, P=1.766e-06). The credible sets from SuSiE-RSS are smaller in size and do not include variants identified by h2-D2 that are seen to be of lower significance than, and at lower levels of LD with, the top variants (see Fig S2.36 of Cordell et al. (2021)). For this reason, we consider the results from SuSiE-RSS to be both the most precise and the most compelling.

Locus 36 (11q23.3)

The top variant from logistic regression is rs11217074 at BP position 118734000 (allele C, β=-0.3744, SE=0.02763, P=7.96e-42). Both SuSiE-RSS and h2-D2 identify a single credible set, of similar size, consistent with previous results from FINEMAP. Based on these results, we consider the results from SuSiE-RSS and h2-D2 to be equally compelling.

Locus 37 (11q24.3)

The top variant from logistic regression is rs10893872 at BP position 128325553 (allele T, β=0.09361, SE=0.02009, P=3.152e-06). The coverage threshold had to be lowered to 0.7 for SuSiE-RSS to identify a single credible set, consistent with previous results from FINEMAP. With this threshold, h2-D2 identifies two credible sets, including variants that seem to have implausibly weak significance levels (minimum P=0.00949). Based on these results, we consider the results from SuSiE-RSS to be the most compelling.

Locus 38 (12p13.31)

The top variant from logistic regression is rs1800693 at BP position 6440009 (allele C, β=0.1761, SE=0.01985, P=7.196e-19). Both SuSiE-RSS and h2-D2 identify a single credible set, of size 1, consistent with previous results from FINEMAP. Based on these results, we consider the results from SuSiE-RSS and h2-D2 to be equally compelling.

Locus 39 (12q24.12)

The top variant from logistic regression is rs10774625 at BP position 111910219 (allele G, β=-0.1859, SE=0.01979, P=5.698e-21). SuSiE-RSS identifies a single credible set of size 3, containing rs10774625, consistent with previous results from FINEMAP. h2-D2 identifies two credible sets, one containing rs1077462 and the other with top SNP rs541707256 at BP position 111927850 (allele TA, β=-0.2090, SE=0.02654, P=3.438e-15). Based on the LD pattern in the region (see Fig S2.40 of Cordell et al. (2021)) we consider the second credible set likely to be part of the first signal. Based on this, along with consistency of SuSiE-RSS's results with FINEMAP, on balance we consider the results from SuSiE-RSS to be the most compelling.

Locus 40 (13q14.11)

The top variant from logistic regression is rs141252748 at BP position 43036726 (allele TG, β=-0.1499, SE=0.01975, P=3.237e-14). Both SuSiE-RSS and h2-D2 identify a single credible set, consistent with previous results from FINEMAP. The credible set from SuSiE-RSS is smaller (20 compared to 38 variants) and does not include variants identified by h2-D2 that are seen to be of lower significance than, and at lower levels of LD with, the top variant (see Fig S2.41 of Cordell et al. (2021)). For this reason, we consider the results from SuSiE-RSS to be both the most precise and the most compelling.

Locus 41 (13q14.2-q14.3)

The top variant from logistic regression is rs9591325 at BP position 50811220 (allele C, β=-0.4408, SE=0.04630, P=1.735e-21). Both SuSiE-RSS and h2-D2 identify a single credible set, of similar size,

consistent with previous results from FINEMAP. Based on these results, we consider the results from SuSiE-RSS and h2-D2 to be equally compelling.

Locus 42 (14q13.2)

The top variant from logistic regression is rs712314 at BP position 35408548 (allele A, β=0.1364, SE=0.02694, P=4.137e-07). Both SuSiE-RSS and h2-D2 identify a single credible set, consistent with previous results from FINEMAP. The credible set from SuSiE-RSS is smaller (13 compared to 136 variants), contains top variant rs712314 (which is not contained in the credible set from h2-D2), and does not include a large number variants identified by h2-D2 that are seen to be of lower significance than, and at lower levels of LD with, the top variant (see Fig S2.43 of Cordell et al. (2021)). For this reason, we consider the results from SuSiE-RSS to be both the most precise and the most compelling.

Locus 43 (14q24.1)

The top variant from logistic regression is rs8008961 at BP position 68752643 (allele T, β=-0.2058, SE=0.02312, P=5.352e-19). h2-D2 identifies a single credible set of size 3 containing rs8008961, consistent with previous results from FINEMAP. SuSiE-RSS identifies two credible sets, one of size 2, containing rs8008961, and one of size 62 containing a large number of SNPs at implausibly low significance levels. Based on these results, we consider the results from h2-D2 as the most compelling.

Locus 44 (14q32.12)

The top variant from logistic regression is rs11624512 at BP position 93111120 (allele T, β=-0.1936, SE=0.02681, P=5.249e-13). Both SuSiE-RSS and h2-D2 identify two credible sets, of similar sizes, consistent with previous results from FINEMAP, one containing rs11624512 and the other whose top SNP is rs10137524 at BP position 93017149 (allele T, β=0.09892, SE=0.02062, P=1.615e-06). Based on these results, we consider the results from SuSiE-RSS and h2-D2 to be equally compelling.

Locus 45 (14q32.32)

The top variant from logistic regression is rs59643720 at BP position 103564807 (allele C, β=0.3252, SE=0.02242, P=1.072e-47). SuSiE-RSS identifies a single credible set, reasonably consistent with previous results from FINEMAP (which had assigned posterior probabilities of 0.39 and 0.54 to 1 and 2 causal variants respectively). h2-D2 identifies five credible sets, one of which includes rs59643720 and the other four that display implausibly low levels of significance (minimum P=0.1589), with results scarcely visible on Supplementary Figure S14. Based on these results, we consider the results from SuSiE-RSS as the most compelling.

Locus 46 (16p13.13)

The top variant from logistic regression is rs12928537 at BP position 11191400 (allele A, β=-0.2403, SE=0.0218, P=3.073e-28). Both SuSiE-RSS and h2-D2 identify two credible sets, consistent with previous results from FINEMAP, one containing rs12928537 and the other whose top SNP is rs243323 at BP position 11361202 (allele G, β=-0.2207, SE=0.02226, P=3.641e-23). The credible sets from SuSiE-RSS are smaller (15 compared to 21, and 6 compared to 10, variants, respectively) and do not include variants identified by h2-D2 that are seen to be of lower significance than, and at relatively

lower levels of LD with, the top variant (see Fig S2.47 of Cordell et al. (2021)). For this reason, we consider the results from SuSiE-RSS to be both the most precise and the most compelling.

Locus 47 (16p12.1)

The top variant from logistic regression is rs1119132 at BP position 27403469 (allele A, β=-0.1942, SE=0.03052, P=1.98e-10). Both SuSiE-RSS and h2-D2 identify a single credible set, of identical size (though containing slightly different variants), consistent with previous results from FINEMAP. Based on these results, we consider the results from SuSiE-RSS and h2-D2 to be equally compelling.

Locus 48 (16q22.1)

The top variant from logistic regression is rs79577483 at BP position 68036939 (allele G, β=0.1962, SE=0.02878, P=9.269e-12). Both SuSiE-RSS and h2-D2 identify a single credible set, consistent with previous results from FINEMAP. The credible set from SuSiE-RSS is smaller (65 compared to 94 variants) and does not include variants identified by h2-D2 that are seen to be of lower significance than, and at lower levels of LD with, the top variant (see Fig S2.49 of Cordell et al. (2021)). For this reason, we consider the results from SuSiE-RSS to be both the most precise and the most compelling.

Locus 49 (16q24.1)

The top variant from logistic regression is rs11117432 at BP position 86019271 (allele A, β=-0.2754, SE=0.02502, P=3.414e-28). Both SuSiE-RSS and h2-D2 identify a single credible set containing the same 2 variants, which is inconsistent with previous results from FINEMAP (which had rather suggested 2 causal variants at this locus, perhaps picking up the more modest signal seen below the top signal). We thus consider the results from SuSiE-RSS and h2-D2 at this locus to be equally compelling.

Locus 50 (17q12)

The top variant from logistic regression is rs8067378 at BP position 38051348 (allele A, β=-0.2690, SE=0.01996, P=2.202e-41). Both SuSiE-RSS and h2-D2 identify a single credible set, of similar size and LD pattern (see Fig S2.51 of Cordell et al. (2021)), consistent with previous results from FINEMAP. Based on these results, we consider the results from SuSiE-RSS and h2-D2 to be equally compelling.

Locus 51 (17q21.31)

The top variant from logistic regression is rs17577094 at BP position 44187492 (allele G, β=-0.1957, SE=0.02532, P=1.077e-14). Both SuSiE-RSS and h2-D2 identify a single credible set, consistent with previous results from FINEMAP. The credible set from h2-D2 is smaller (746 compared to 1909 variants) and seems more consistent with the LD pattern in the region (see Fig S2.52 of Cordell et al. (2021)). For this reason, we consider the results from h2-D2 to be both the most precise and the most compelling.

Locus 52 (18q22.2)

The top variant from logistic regression is rs17207042 at BP position 67537351 (allele T, β=0.1228, SE=0.01976, P=5.164e-10). Both SuSiE-RSS and h2-D2 identify a single credible set, of identical size and similar LD pattern (see Fig S2.53 of Cordell et al. (2021)), consistent with previous results from

FINEMAP. Based on these results, we consider the results from SuSiE-RSS and h2-D2 to be equally compelling.

Locus 53 (19p13.2)

The top variant from logistic regression is rs34725611 at BP position 10477067 (allele G, β=-0.2032, SE=0.02260, P=2.398e-19). Both SuSiE-RSS and h2-D2 identify a single credible set containing the same 2 variants, consistent with previous results from FINEMAP. We thus consider the results from SuSiE-RSS and h2-D2 at this locus to be equally compelling.

Locus 54 (19p13.11)

The top variant from logistic regression is rs1811241 at BP position 18235882 (allele A, β=-0.1289, SE=0.02164, P=2.543e-09), which is identified by h2-D2. SuSiE-RSS does not identify rs1811241; instead the top variant identified by SuSiE-RSS is rs72999449 at BP position 18237050 (allele T, β=-0.134, SE=0.02338, P=9.91e-09). Both SuSiE-RSS and h2-D2 identify a single credible set, consistent with previous results from FINEMAP. Although the credible set from SuSiE-RSS is more precise (23 as compared to 34 variants), and does not include two variants identified by h2-D2 that have low levels of significance (P=1.054e-05 and P=4.936e-05), the fact that SuSiE-RSS misses rs1811241 (whereas it is identified in the credible set from h2-D2) leads us to consider the results from h2-D2 to be most compelling.

Locus 55 (19q13.33)

The top variant from logistic regression is rs3745516 at BP position 50926742 (allele A, β=0.2721, SE=0.02231, P=3.276e-34). Both SuSiE-RSS and h2-D2 identify two credible sets, of similar sizes, consistent with previous results from FINEMAP, one containing rs3745516 and the other whose top SNP is rs35228262 at BP position 50909107 (allele A, β=-0.2001, SE=0.04524, P=9.702e-06). Based on these results, we consider the results from SuSiE-RSS and h2-D2 to be equally compelling.

Locus 56 (22q13.1)

The top variant from logistic regression is rs137687 at BP position 39740078 (allele A, β=-0.2140, SE=0.02029, P=5.300e-26). Consistent with previous results from FINEMAP (posterior probability 0.59), h2-D2 identifies two credible sets, one containing rs137687 and the other whose top SNP is rs138384476 at BP position 39707781 (allele T, β=-0.1124, SE=0.03329, P=0.0007359). Slightly less consistent with previous results from FINEMAP (posterior probability 0.41), SuSiE-RSS identifies three credible sets, two of which correspond to the credible sets from h2-D2 and the third of which contains number of SNPs at implausibly low significance levels (minimum P=0.1850). Based on these results, we consider the results from h2-D2 as the most compelling.

References

Cordell, H. J., Fryett, J. J., Ueno, K., Darlay, R., Aiba, Y., Hitomi, Y., Kawashima, M., Nishida, N., Khor, S.-S., Gervais, O., et al. (2021). An international genome-wide meta-analysis of primary biliary cholangitis: Novel risk loci and candidate drugs. *Journal of Hepatology*, 75(3):572–581.
